# Composition and in situ structure of the *Methanospirillum hungatei* cell envelope and surface layer

**DOI:** 10.1126/sciadv.adr8596

**Published:** 2024-12-13

**Authors:** Hui Wang, Jiayan Zhang, Shiqing Liao, Anne M. Henstra, Deborah Leon, Jonathan Erde, Joseph A. Loo, Rachel R. Ogorzalek Loo, Z. Hong Zhou, Robert P. Gunsalus

**Affiliations:** ^1^ Department of Microbiology, Immunology, and Molecular Genetics, University of California, Los Angeles (UCLA), Los Angeles, CA 90095, USA.; ^2^ Department of Bioengineering, UCLA, Los Angeles, CA 90095, USA.; ^3^ California NanoSystems Institute, UCLA, Los Angeles, CA 90095, USA.; ^4^ Department of Chemistry and Biochemistry, UCLA, Los Angeles, CA 90095, USA.; ^5^ UCLA-DOE Institute, UCLA, Los Angeles, CA 90095, USA.

## Abstract

Archaea share genomic similarities with Eukarya and cellular architectural similarities with Bacteria, though archaeal and bacterial surface layers (S-layers) differ. Using cellular cryo–electron tomography, we visualized the S-layer lattice surrounding *Methanospirillum hungatei*, a methanogenic archaeon. Though more compact than known structures, *M. hungatei*’s S-layer is a flexible hexagonal lattice of dome-shaped tiles, uniformly spaced from both the overlying cell sheath and the underlying cell membrane. Subtomogram averaging resolved the S-layer hexamer tile at 6.4-angstrom resolution. By fitting an AlphaFold model into hexamer tiles in flat and curved conformations, we uncover intra- and intertile interactions that contribute to the S-layer’s cylindrical and flexible architecture, along with a spacer extension for cell membrane attachment. *M. hungatei* cell’s end plug structure, likely composed of S-layer isoforms, further highlights the uniqueness of this archaeal cell. These structural features offer advantages for methane release and reflect divergent evolutionary adaptations to environmental pressures during early microbial emergence.

## Teaser

Cryo–electron tomography of the cylindrical archaeal cell resolves the surface layer structure and subunit interactions.

## INTRODUCTION

Archaeal cells have distinct morphological features compared to Bacteria and Eukarya. They use a unique cell envelope with differences in overall topology, molecular composition, and evolutionary origin. First, archaeal cells lack the membrane-bound nucleus and cytoplasmic membranes containing fatty acids found in eukaryotic cells (*1*). Second, the archaeal surface layer (S-layer) bears no resemblance to the cell walls of plants, fungi, and algae found in Eukarya. Third, unlike most bacterial cell walls, archaeal S-layers rarely contain peptidoglycans (*2*), and their phospholipid membranes use ether-linked phytanoyls of C20 and/or C40 chain length instead of fatty acids and cholesterol found in Bacteria and Eukarya membranes (*1*, *3*).

*Methanospirillum hungatei* JF1, a model archaeal species, was first isolated from municipal wastewater where it works with bacteria and fungi to recycle complex organic matter into methane, water, and carbon dioxide (*4*). *M. hungatei* cells are curved to spiral shaped rods, ~0.4 to 0.5 μm in diameter and ~7 μm long, with polar flagella that enable motility and taxis (*4*–*6*). Its cytoplasmic membrane is surrounded by a proteinaceous S-layer that functions similarly to the peptidoglycan-containing cell walls of Gram-positive and Gram-negative bacteria (*7*–*11*). In addition, the outer sheath layer encapsulates multiple *M. hungatei* cells to form chains of 2 to more than 70 cells, reaching lengths of up to 500 μm (*4*, *12*).

The cell envelopes of Archaea play critical roles in prokaryotic physiology, affecting cell size and shape and providing protection from environmental agents and phages (*13*). Since the initial observation of S-layers more than 50 years ago (*14*), electron microscopy has revealed their low-resolution organization (*15*–*18*). Early x-ray crystallography efforts from recombinantly expressed tandem repeat of *Geobacillus stearothermophilus* (*19*) and *Methanosarcina acetivorans* (*20*) surface layer proteins (SLPs) have revealed their β-sandwich domains. Recent advances in cryo–electron tomography (cryo-ET) have enabled high-resolution, three-dimensional (3D) in situ analyses of SLPs in *Caulobacter crescentus* (*21*, *22*), *Haloferax volcanii* (*23*), *Deinococcus radiodurans* (*24*), *Sulfolobus acidocaldarius* (*25*), and *Nitrosopumilus maritimus* (*26*) showing hexagonal tiled lattices coating and protecting the cell membrane with distinctive subunits from bacterial and archaeal lineages. Despite these advancements, the native arrangements and conformations of S-layer repeats remain poorly understood, as does their role in cell division.

In this study, we identified the *M. hungatei* SLP, Mhun_2263, and determined the in situ structure of the S-layer hexamer tile at 6.4-Å resolution using cellular cryo-ET and subtomogram averaging (STA). Guided by the resolved secondary structure and tertiary fold, we fitted the SLP monomer atomic model from AlphaFold2 (*27*, *28*) and established the molecular interactions essential for SLP assembly into hexamers. We captured the SLP hexamer in both flat and curved states, enabling the investigation of domain rearrangement necessary for the formation of a semiflexible hexagonal lattice enclosing the cell. Our structures also revealed S-layer contacts with the outer sheath layer and the underlying cell membrane, elucidating its anchoring and assembly. In addition, we uncovered the complete architecture of enveloped *M. hungatei* cells, resolving the multilayered end plugs and protruding flagella and pili within the encapsulated cell ends.

## RESULTS

### Resolving the S-layer architecture of *M. hungatei* by cellular cryo-ET

**Fig. 1.**
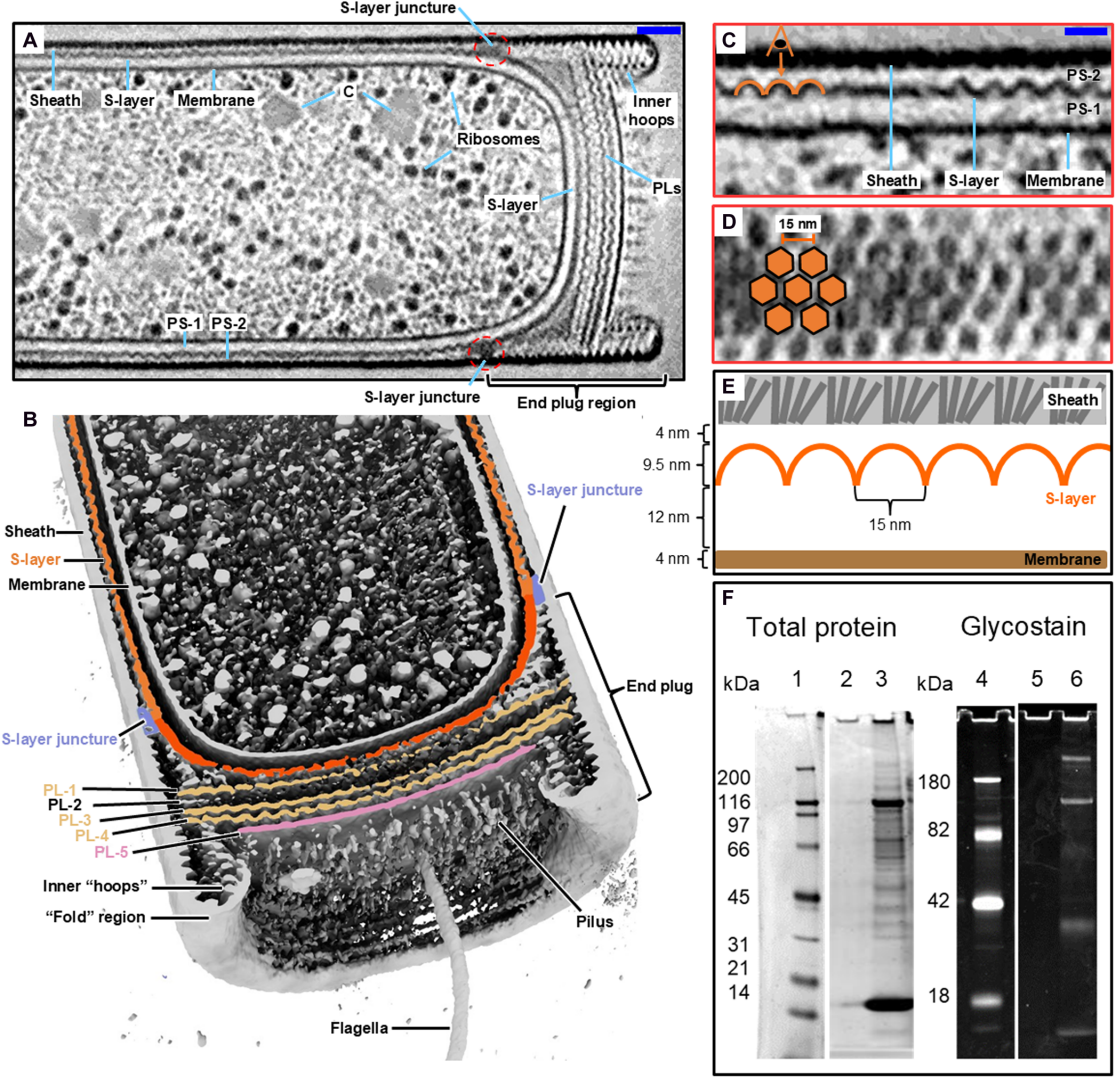
Overview of the *Methanospirillum hungatei* S-layer. (**A**) Tomographic slicer view of a *M. hungatei* cell near the end plug region, showing S-layer sandwiched between the cell membrane and the outermost sheath layer. Intracellular structures, such as ribosomes and membrane-less condensate (C), can be recognized inside the cell. Scale bar, 50 nm. (**B**) Iso-surface of one tomogram of a *M. hungatei* cell near the end plug region, with a cross section showing labeled major components. (**C** and **D**) Orthogonal slicer views of the zoom-in S-layer structure, showing its hexagonal assembly pattern. The viewing angle in (D) is indicated in (C). Scale bar, 20 nm. (**E**) A schematic diagram showing the typical dimensions and topology of the *M. hungatei* cell envelope. (**F**) Identification of surface labeled *M. hungatei* proteins by SDS-PAGE, showing the major S-layer glycoprotein with a molecular weight of 116 kDa. Lane 1, SYPRO-Ruby-stained protein ladder; lane 2, final bead wash; lane 3 bead eluate; lane 4, Candy Cane protein ladder; lane 5, final bead wash; lane 6, bead eluate.

To examine the organized structural complexity of the *M. hungatei* envelope at higher resolution, we captured in situ cell images using cryo-ET (Fig. 1, A to D). Reconstructed tomograms reveal its envelope consisting of a proteinaceous S-layer sandwiched between the cell lipid membrane and an outermost sheath layer, forming periplasmic spaces (PS) termed PS-1 and PS-2, respectively (Fig. 1, A to C). The S-layer densities, appearing as approximate waves in its side view, contrasting with the sheath layer (Fig. 1C), and its repeated hexagonal lattice can be resolved from the top view, suggesting a hexagonal arrangement of subunits (Fig. 1D). The initial STA of the S-layer tiling subunit, without imposing symmetry, suggested a sixfold symmetry arrangement (fig. S1). The S-layer lattice completely surrounds the cytoplasmic membrane of individual cells to enclose PS-1 while being spatially constrained by the outermost sheath layer and circular end plugs with diameters of ~400 nm located between *M. hungatei* cells (*29*). With a height of ~9.5 nm and a unit (center to center) distance of ~15 nm, the hexagonal S-layer lattice maintains a uniform 4-nm space from its cap domain to the sheath layer and a 12-nm space from its domain base to the cell lipid membrane (Fig. 1E), over the entire length of cells except at the ends where distinct plug elements are positioned.

We also captured the in situ *M. hungatei* cell end plug region, which is shaped like a soda can lid to enclose the cell (Fig. 1, A and B). The end plug contains five stacked round plug layers (PLs), which share a similar diameter but vary in shape. The outermost layer (PL-5) is exposed to the extracellular environment and is relatively flat, while PL-1, PL-3, and PL-4 are wavy, and PL-2 is composed of weak densities with “Y-shape” subunits bridging PL-1 and PL-3 (fig. S2). Those wavy layers (PL-1, PL-3, and PL-4) resemble the S-layer but have a smaller height of ~8 nm and a larger hexagonal unit distance of ~18 nm (fig. S2). The sheath tube extends beyond the PLs and connects to an inner tube-like structure consisting of smaller hoops (*29*), via a folded density at the tip to form an extruding rim-shaped structure and seals the cell together with the abovementioned PLs (Fig. 1, A and B, and fig. S2). The S-layer at the end plug region also becomes thicker and transitions from wavy to a more gently curved surface after passing the dense S-layer juncture (Fig. 1A), possibly to support the local synthesis of sheath layer, end plugs, S-layer, and cell appendages (i.e., flagella and pili).

### Identification of the *M. hungatei* SLP

Next, we identified the *M. hungatei* cell SLPs using an in vivo cell surface biotinylation labeling strategy (*30*), as detailed in Materials and Methods. Following cell labeling, protein extraction, and affinity purification, a major ~116-kDa species was observed by SDS–polyacrylamide gel electrophoresis (SDS-PAGE) (Fig. 1F, lane 3) that appears to be posttranslationally modified, as revealed by glycoprotein staining (Fig. 1F, lane 6). The two smaller bands correspond to the *M. hungatei* flagella and pili (*31*). Liquid chromatography–tandem mass spectrometry (LC-MS/MS) analysis identified two nearly identical proteins (Mhun_2263 and Mhun_2513) in the larger band (table S1), each highly abundant in intact cells. These two SLP paralogs share ~88.5% amino acid sequence identity and both have a ~216–amino acid domain of the InterPro-dubbed “domain of unknown function”, DUF3821 (fig. S3), which is exclusively found within a subgroup of methanogenic archaea (i.e., the Methanomicrobiales) (*32*). However, Mhun_2263 contains an additional conserved PGF-CTERM domain (fig. S3A), termed pfam18204/IPR026371, which is predicted to direct posttranslational proteolytic processing with subsequent lipid attachment to anchor it in the cytoplasmic membrane (*33*, *34*).

The AlphaFold model of Mhun_2263 suggests that the SLP monomer is composed of six immunoglobulin (Ig)–like domains (D1 to D6) connected by relatively flexible linkers, followed by a ~50–amino acid spacer and the PGF-CTERM domain (fig. S3B). The multidomain arrangement of the monomer shows high similarity to that of the *H. volcanii* SLP (*23*) (fig. S4A), despite their relatively low-sequence identity (fig. S4B). D1 and D2 together constitute DUF3821, and the corresponding sequence is distinctively different from and 50 amino acids longer than that of the *H. volcanii* SLP (fig. S4B and table S2). Consequently, the sizes of D1 and D2 are larger in *M. hungatei* (fig. S4A). Additional properties of the *M. hungatei* SLP paralogs (Mhun_2263 and Mhun_2513) and *H. volcanii* Hvo_2072 are summarized in tables S1 and S2.

### The SLP hexamer resolved by cryo-ET STA

**Fig. 2.**
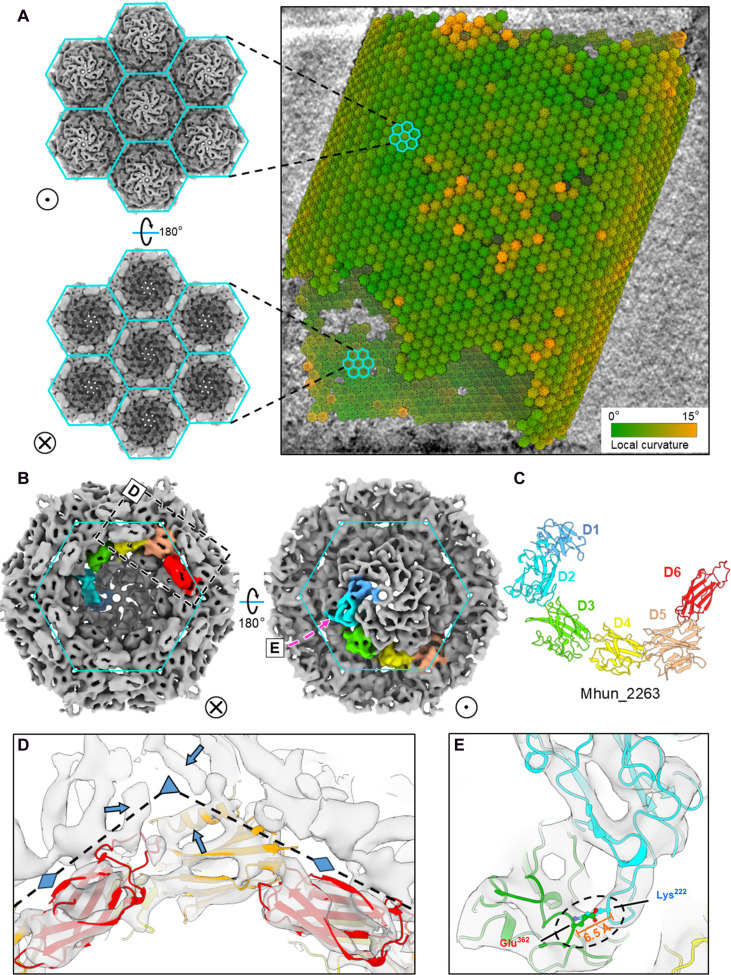
The SLP hexamer STA result and its modeling with AlphaFold. (**A**) The S-layer lattice of a *M. hungatei* cell, represented by placing back averaged hexamers into the original tomogram. Color represents the local lattice curvature. The exterior and interior viewing directions are indicated by a dot in a circle and a cross in a circle, respectively, and these two indicators apply in all other figures. (**B**) The STA result of S-layer hexamer with one SLP monomer is colored by Ig-like domains. The cyan outline encloses one S-layer hexamer tile. The dashed box encloses the region shown in (D), and the pink arrow points to region shown in (E). (**C**) The SLP monomer model of Mhun_2263 was adapted from AlphaFold prediction by individually rigid-body fitting of domains D1-D6. (**D** and **E**) Docking the AlphaFold model into the SLP hexamer map shows matches of Ig-like domains and an α helix at the trimeric interhexamer interface (indicated by blue arrows). The STA result also reveals an interdomain connection potentially formed by Lys^222^ and Glu^362^. Triangle and diamond shapes indicate the trimeric and dimeric interhexamer interfaces, respectively.

To resolve structural details of the S-layer building block, we next obtained an STA C6 map at 6.4-Å resolution with the interhexamer interfaces well resolved (Fig. 2, A to C, fig. S5A, and movie S1). The main body of the STA map is a dome-shaped hexagonal density, dotted by domains of β sheet–like convex surfaces characteristic of Ig-like folds, joined by linking densities (Fig. 2B and fig. S6A). Guided by the STA map, the AlphaFold-predicted Ig-like domains were rearranged by rigid-body fitting into the corresponding densities, leading to a pseudoatomic model of the *M. hungatei* SLP monomer (Fig. 2, B and C), which has a sickle shape similar to that of the *H. volcanii* SLP (*23*). Each domain features an empty space between the two β sheets of the Ig-like domains (Fig. 2, D and E), and the one next to the trimeric interhexamer interface has a rod-shaped α helix (indicated by the arrows in Fig. 2D). Notably, the fitting also shows that all densities of the STA map are fully accounted with a model-to-map cross-correlation coefficient of ~0.7, and there is no visible model overlap between interacting domains, either within a hexamer or between neighboring hexamers.

**Fig. 3.**
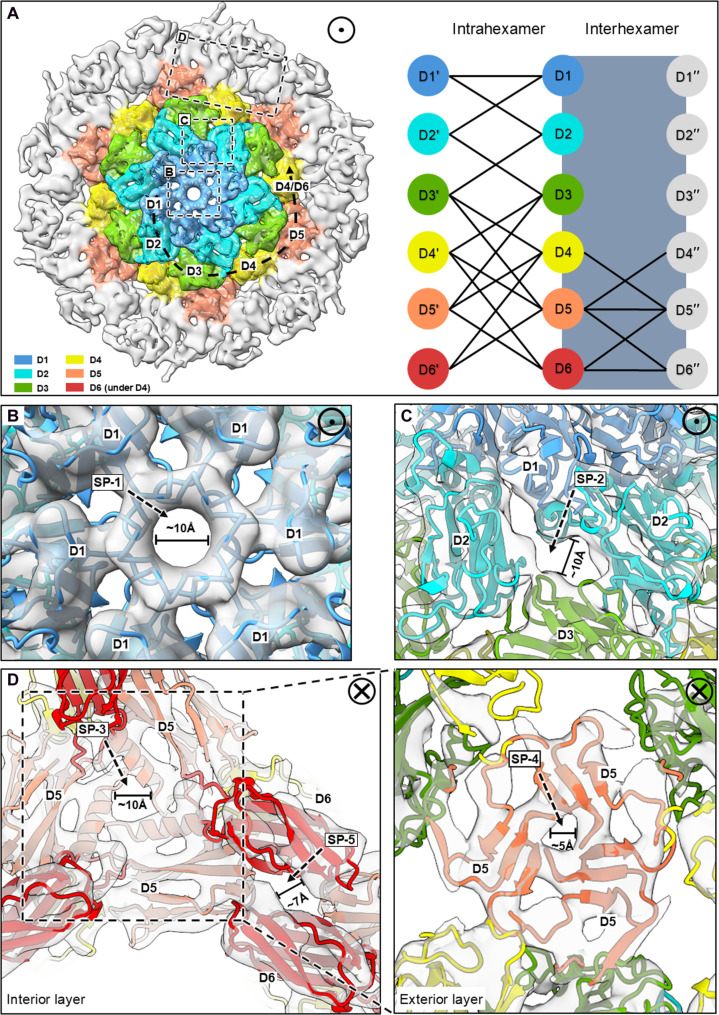
Intra- and interhexamer domain interactions and S-layer pores. (**A**) Left: A guide map with D1-D6 colored differently in a SLP hexamer. Dashed boxes indicate the locations of S-layer pores (SP-1 to −5) as shown in (B) to (D). Right: A diagram of intra- and interhexamer contacts between D1-D6, showing that only D4-D6 are involved in establishing interhexamer connections, with D5 serving as an interaction hub. (**B**) SP-1 is at the hexameric intrahexamer interface maintained by D1 domains. (**C**) SP-2 is at the intrahexamer interface between adjacent domains D1-D2 and D2-D3. (**D**) SP-3 and SP-4 (inset) are at the trimeric interhexamer interface, maintained by contacting α helices and β sheets, respectively, from D5 domains. SP-5 is at the dimeric interhexamer interface.

The *M. hungatei* SLP hexamer forms a lattice through extensive interactions between Ig-like domains, where D1, D2, and D3 participate solely in intrahexamer assembly, while D4, D5, and D6 participate in both intra- and interhexamer interactions (Fig. 3A and fig. S6, B to E). Varying in size, shape, and location, multiple S-layer pores (here termed SP-1 to SP-5) have sufficient sizes (Fig. 3, A to D, and fig. S7) to support entry and exit of small molecules to function in material exchange (*35*). The subunits of *M. hungatei* SLP hexamer are more compactly assembled as compared to those in other archaeal species. For example, *M. hungatei* shares a similar SLP monomer and hexamer shape/topology with *H. volcanii* but has larger SLP subunit domains (i.e., D1 and D2) (fig. S4A), yet both height and diameter of the *M. hungatei* SLP hexamer are shorter by 5 and 20 Å, respectively (fig. S8A). As a result, the S-layer pores appear narrower in *M. hungatei* (fig. S8A).

### Tiling SLP hexamers into the characteristic “cylinder-like” lattice

**Fig. 4.**
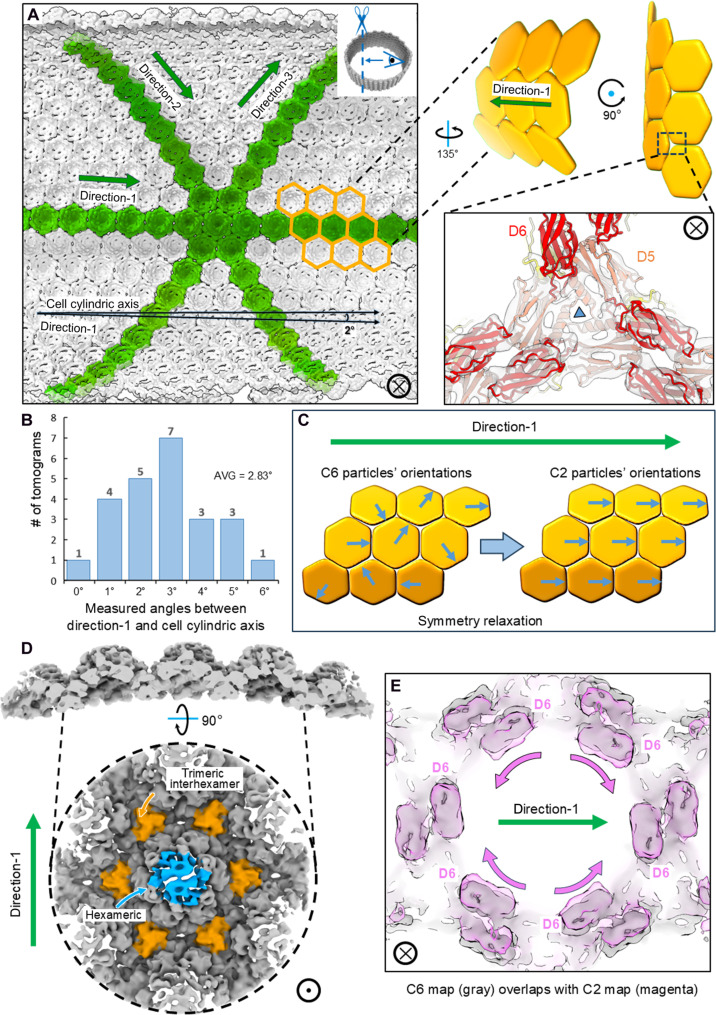
The SLP hexamer in a curved conformation. (**A**) Interior view of the S-layer lattice showing the tiling of SLP hexamers in three directions (termed as direction-1, direction-2, and direction-3), with direction-1 running along the cylindrical axis of the cell. Inset: A diagram of 9 SLP hexamers, each represented by a rigid hexagon shape, indicating the adaptation of interhexamer interfaces required for the formation of cylindrical curvature. (**B**) Histogram of measured angles in degrees between direction-1 and the cylindrical axis of the cell among 24 *M. hungatei* cells, with an average angle of 2.83°. (**C**) Illustration of symmetry relaxation from C6 to C2 along direction-1. (**D**) The STA result of the SLP hexamer in a curved conformation, with its hexameric interface colored blue and trimeric interhexamer interface colored orange. (**E**) Overlap of the C6 and C2 SLP hexamer STA maps showing the movement of dimeric interhexamer interfaces that are not along direction-1.

The S-layer lattice of the cylindrical *M. hungatei* cell appears compressed during sample vitrification, becoming elliptical—flattened near the air-water interface and more curved along the sides away from it (Fig. 2A). The SLP hexamers tile into a hexagonal lattice along three directions off-set by 60°, with each direction defined by the vector connecting the centers of two adjacent hexamers (direction-1, direction-2, and direction-3 in Fig. 4A). Notably, one tiling direction runs along the cylindrical axis of the cell (direction-1 in Fig. 4, A and B), while the other two curve around the cylinder (Fig. 4A), and this tiling pattern is also found in other archaeal species with similar S-layer shapes (*26*). This arrangement flattens the dimeric interhexamer interface along the straight direction (i.e., direction-1), while twisting/tilting along the curved tiling directions (i.e., direction-2 and direction-3) to accommodate the cylinder’s curvature (top inset of Fig. 4A).

Taking advantage of the S-layer tiling pattern, we performed STA on particles only from curved portions of the S-layer lattice and applied C2 symmetry along direction-1 to capture the SLP hexamer structure in the curved conformation (Fig. 4, C and D). The trimeric D5 interhexamer interface, known to be essential for the S-layer lattice assembly (*23*, *36*), is maintained by the combination of an α helix and a three–β strand sheet in *M. hungatei* (Figs. 3D and 4D and movie S2), but a conserved α-hairpin in haloarchaea (*23*). Comparing the flat and curved lattice from STA of C6 and C2 reconstructions, the trimeric D5 interhexamer interface remains the same size and shape to maintain lattice connectivity, while the hexameric interface and dimeric interhexamer interfaces show substantial conformational changes (Fig. 4, D and E). The morph between the predicted atomic models, built from STA of C6 and C2 reconstructions, shows the rearrangement of Ig-like domains to adapt to the curvature and indicates that D1, D5, and D6 have larger movements (table S3 and movie S3), as they are essential to the formation of hexameric intrahexamer, trimeric interhexamer, and dimeric interhexamer interfaces, respectively. The two negatively charged surfaces at opposing sides at the dimeric interhexamer interface (fig. S9) would repel each other, explaining the distinctive gap at that interface and potentially providing flexibility for the observed lattice curvature.

### Connections from S-layer to sheath layer and from S-layer to cell membrane

The uniformity in both periplasmic-like spacings (i.e., PS-1 and PS-2) implies a mechanism to control layer positioning. While the cryo-ET densities are of insufficient resolution to resolve structures spanning these spaces, the integration of cryo-ET structures, mass spectrometry data, and AlphaFold models offers plausible explanations.

**Fig. 5.**
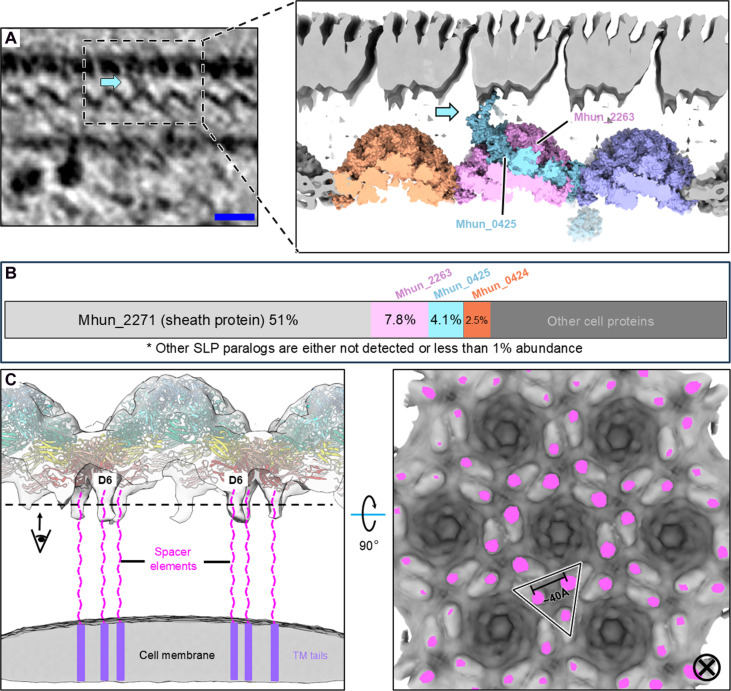
The *M. hungatei* S-layer is uniformly spaced from the sheath and cell membrane. (**A**) Tomographic slicer view of the *M. hungatei* envelope showing the observed connection between the S-layer and sheath layer. Inset: A proposed model depicting a mixed hexamer of Mhun_2263 and Mhun_0425. The cyan arrow indicates the connection density. Scale bar, 20 nm. (**B**) Abundance distribution diagram of sheath-associated proteins, showing the presence of three SLP paralogs including Mhun_2263, Mhun_0425 and Mhun_0424. (**C**) Left: Proposed model of S-layer anchoring to the cell membrane through the C-terminal spacer and PGF-CTERM domains. Right: Cross-sectional view of the density map at lower threshold showing the spatial arrangement of the spacers (colored magenta) extending from D6 domains.

First, in the cellular tomograms, we observed densities connecting some of the S-layer cap regions and the sheath layer (Fig. 5A), suggesting that additional proteins are recruited to establish this contact and thus maintain PS-2. An analysis of enriched sheath preparation by LC-tandem-MS revealed the presence of two other sheath-associated SLP paralogs (Mhun_0425 and Mhun_0424) (table S4), at lower abundance relative to Mhun_2263 and the sheath protein, Mhun_2271 (Fig. 5B). The AlphaFold model of Mhun_0425 displays analogous Ig-like domains D1 to D5, plus additional Ig-like and unstructured coil domains located at both N and C termini (fig. S10 and table S5). This inspired us to model an SLP hetero-hexamer with five Mhun_2263 and one Mhun_0425 (Fig. 5A, inset). The additional N-terminal Ig-like domain with a coil extension could expand across ~4 nm into the PS-2 region and contact the sheath layer.

Second, we also observed filament-like densities extending from each D6 domain (Fig. 5C), which account for the C-terminal spacers that anchor to the cell membrane with PGF-CTERM domains, thus maintaining PS-1 spacing. When cells are subjected to osmotic shock, the outer sheath layer delaminates from the S-layer, while the PS-1 spacing is still maintained (fig. S11), suggesting that stronger interactions maintain the latter. The proline-rich 50–amino acid spacers extend from the connected D6 domains and space ~40 Å from the closest ones with no tendency to combine (Fig. 5C), suggesting they are not able to form a trimeric coiled coil used in *S. acidocaldarius* (*25*) for membrane anchoring. Thus, they should reach cell membrane independently and together maintain the uniform 12-nm spacing of PS-1.

## DISCUSSION

This study establishes the overall 3D architecture of the *M. hungatei* cell envelope and offers insight into the composition and assembly of its associated S-layer. The *M. hungatei* S-layer adapts to the cylindered shape of its external sheath layer to encapsulate its cell membrane, distinguishing it from the globular or pleomorphic shapes of S-layers in most other archaeal cell types (*37*). One posttranslationally modified SLP, Mhun_2263, was identified that forms the dome-like hexameric S-layer tile structure, with its PGF-CTERM domain likely anchored to the cell membrane. Fitting the AlphaFold models into the resolved in situ SLP hexamer structures reveals interactions among the various Ig-like domains that are important to the lattice assembly and elucidates the rearrangements among these Ig-like domains that enable the asymmetric curvature.

### Architecture and permeability of the *M. hungatei* S-layer

*M. hungatei* has a special need to allow the exhalation of methane gas as compared to other nonmethanogenic microbes. It uses a continuous but perforated S-layer sheet to achieve permeability between the inside and outside of the cell, in contrast to the strategy used by Gram-negative bacteria, which have protein channels made up of β barrel structures embedded in their outer membranes. At the architectural level, the characteristic cylindrical shape of *M. hungatei* cells doubles the surface area–to–volume ratio compared to that of a sphere with the same volume (*29*). In addition, the *M. hungatei* S-layer hexamer tile, with its dimpled “egg carton” or “waffle-like” topology and a height of 95 Å, results in an ~30 to 40% higher exposed surface area than that of a flat surface. At the molecular level, the *M. hungatei* SLP hexamer has distinct pore types that together would provide passage conduits for small molecules, such as nutrients, methane gas, and waste products (fig. S7). Each pore is of sufficient size (5 to 10 Å) to allow the movement of hydrogen, carbon dioxide, and acetate substrates as well as essential minerals, from PS-2 to the cytoplasmic membrane. The major *Borrelia burgdorferi* P13 outer membrane porin, with its small 1.4-Å channel, is sufficient to allow transit of molecules of 400-Da molecular mass (*38*). The number of pores present within one S-layer hexamer tile is seven (one of type SP-1 and six of SP-2), with an additional five pores on average formed by junctures of adjoining tiles (two for SP-3 and SP-4, and three for SP-5). One *M. hungatei* cell, with a cylindrical shape of 0.4-μm radius and 7-μm length, contains ~268,416 SLP monomers or ~44,736 SLP hexamers, based on the calculation with 466 and 96 repeats along the cell cylinder axis and circumference, respectively. Correspondingly, the number of S-layer pores would be ~536,832 per cell, with an average of 12 per hexamer.

A challenge to the *M. hungatei* cell arising from using cylindrical other than spherical cellular architecture is how to close the two ends of the cylinder. Our cryo-ET structure reveals the architectural arrangement of the end plug region and its associated flagella plus pili in higher detail than previously reported (*39*, *40*). An inward folded region seals and presumably retains the multilayered end plugs, separating the encapsulated cells within the communal sheath (*29*). Yet, unknown is the molecular identity and structure of these layered end plugs that partition and space individual cells. These five clearly resolved layers, with distinct shapes and thickness (fig. S2), are now revealed with flagella and pili threaded across the plug. Cell motility would be driven by an archaeal adenosine triphosphate–dependent motor emanating from the cell ends and controlled by multiple membrane and cytoplasmic chemoreceptors (methyl-accepting chemotaxis proteins) (*41*, *42*). As the flagella and pili are anchored only in the polar region, other than those in Bacteria, the overall cylindrical shape also offers the necessary directionality for motility.

The intact S-layer lattice forms a cylindrical tube between two cell end plugs, which close the cell envelope. The usual strategy to bring a hexagonal lattice to closure is the utilization of pentamers, exemplified by icosahedral virus capsid (*43*), cone-shaped HIV capsids (*44*), and S-layer lattices in both pleomorphic (*23*) and cylindrical (*26*, *45*) cells. For example, in *Methanoregula formicica*, the insertion of pentamers leads to vertices and sharp edges in the S-layer and cell membrane (fig. S12, A and B). By rearranging Ig-like domains, the SLP hexamers can form a tube shape without involving pentamers. No SLP pentamers were observed in the tube region of the S-layer. However, after passing through the S-layer juncture structure, the S-layer sheet become thicker and bend inward to seal the cell end in the end plug region (Fig. 1, A and B), where vertices and sharp edges in the S-layer and cell membrane caused by pentamer insertion were observed (fig. S12C), suggesting a similar strategy for envelop closure.

### SLP orthologs and paralogs

More than 300 *M. hungatei* JF1 S-layer orthologous proteins have been identified within other methanogenic archaea, including strains of the following 10 genera: *Methanospirillum*, *Methanoregula*, *Methanoculleus*, *Methanofollis*, *Methanoplanus*, *Methanogenium*, *Methanomicrobium*, *Methanocacinia*, *Methanolina*, and *Methanospaerula.* No orthologs were found in other archaeal Euryarchaeota or in any bacteria species based on BLAST comparison or by the presence of the DUF3821 signature. Notably, the shared Ig-like domain structure is a common theme, although this theme represents a highly diverse group of heterogeneous structures. The *M. hungatei* SLP monomer forms a hexagonal lattice structure, similar to those described in *M. acetivorans* (*20*), *H. volcanii* (*23*), *C. crescentus* (*21*), *S. acidocaldarius* (*25*), and *N. maritimus* (*26*). These structures differ in size, sequence, and domain structure, as well as lattice dimensions and porosity. While the *M. hungatei* and *H. volcanii* exhibit similar S-layer topology, they appear to be of divergent structural, phylogenetic, and environmental origins.

Two nearly identical paralogous SLPs, Mhun_2263 and Mhun_2513, exist abundantly in intact *M. hungatei* cells, with Mhun_2263 containing an additional PGF-CTERM (also termed pfam18204/IPR26371). There are 14 SLP paralogs encoded in the *M. hungatei* JF1 genome that vary in size, domain structure, and gene location (table S4). The structural roles for all these proteins are currently unknown, but two (Mhun_0425 and Mhun_0424) were detected in enriched sheath preparations besides Mhun_2263. In addition to the conserved Ig-like domains D1-D5, Mhun_0425 has an ~100–amino acid N-terminal extension; therefore, hetero-hexamers comprising of Mhun_2263 and Mhun_0425 subunits could be used to establish contacts with both the sheath layer and the cell membrane. Notably, Mhun_2513 was also detected in sheath preparations when CHAPS detergent was omitted.

### Biogenesis of the *M. hungatei* S-layer

The pathway for S-layer biogenesis in *M. hungatei* is unknown but would be coordinated to synchronize with cell growth and division by a genetically predetermined temporal and spatial program. The extracellular location of the S-layer is consistent with an S-layer synthesis and assembly model similar to that put forth for the sheath layer (*29*). Sheath precursors are synthesized in the cytoplasm, exported across the cell membrane near the cell ends, and then inserted into the elongating sheath structure at transient gaps. Likewise, the synthesis, glycosylation, and transmembrane translocation of the SLP monomer could occur near the cell ends. Specifically, we suggest that SLP synthesis, sorting, and insertion into the S-layer occur near the “S-layer juncture,” which is a density anchor to the sheath where the S-layer extends on one side of the cell body into the cylinder and on the other side curves and flattens at the end plugs (Fig. 1, A and B). These S-layer juncture structures could coordinate the insertion of nascent translated SLPs from the cytoplasm, the formation of intra- and intertile contacts unveiled in this study, and the assembly of both the cylindrical S-layer tube and end plugs.

## MATERIALS AND METHODS

### Sample preparation and cryo-ET tilt-series acquisition of *M. hungatei* cells

*M. hungatei* strain JF1 (American Type Culture Collection 27890) was cultured anaerobically with a vessel headspace pressurized to 10 psi (68,947.57 pascals) with an 80:20 (vol/vol) mixture of H_2_:CO_2_ as described previously (*29*, *31*). Cells were serially transferred at least three times, with transfers made at mid-exponential phase, to achieve 10+ cell doublings before harvest.

Harvested *M. hungatei* cells were mixed with fiducial gold beads of 5 nm in diameter. The mixture (3 μl) was applied onto Quantifoil (3:1) holey carbon grids that were freshly glow discharged for 30 s at −40 mA. With an FEI Mark IV Vitrobot, excess sample on the grid was blotted away with filter paper at a blot force of −4 and blot time of 5 s. The sample was vitrified immediately by being plunged into liquid nitrogen–cooled liquid ethane. Plunge-freezing conditions and cell concentration on the grids were optimized with an FEI T20 transmission electron microscope equipped with an Eagle 2 K HS CCD camera. Grids with vitrified cells were stored in a liquid nitrogen dewar until use. With either FEI Batch Tomography (lower magnification dataset, with Volta phase plate) or SerialEM (*46*) (higher magnification data that used for STA), tilt series were collected in a Titan Krios instrument equipped with a Gatan imaging filter (GIF) and a post-GIF K2 direct electron detector in electron-counting mode.

### Cryo-ET image processing, 3D reconstruction and STA

The cryo-ET and STA data processing was performed using TomoNet (*47*) together with Relion4 (*48*). Raw frames in each movie were drift corrected with Motioncorr2 (*49*), which produced a single micrograph for each tilting angle. Each tilt series, consisting of stacked tilted micrographs, was reconstructed into 3D tomograms using the IMOD software package (*50*). Under four-binned pixel size, tomograms were processed using IsoNet (*51*) to perform missing wedge correction and to improve the contrast for facilitating particle picking.

To improve the signal-to-noise ratio and enhance the resolution, STA was applied to SLP subunits, and “Auto Expansion” function was used to pick SLP building blocks on the S-layer lattice. After particle picking, an initial reference map was generated using PEET program (*52*) without imposing symmetry (fig. S1). Using “Other Utilities” in TomoNet, the particles’ information, including their coordinates and initial orientations, was imported into a STAR file following Relion4’s convention. Following one round of “3D auto-refine” under four-binned pixel size and two rounds of “3D auto-refine” under two-binned and original pixel sizes, along with the removal of “bad” particles by 3D classification in TomoNet using geometric constraints, the resulting resolution for the C6 SLP hexamer structure is 6.4 Å. To obtain the curved conformation of the SLP hexamer, symmetry relaxation was performed on the particles only at the curved portion of the S-layer lattice (Fig. 5C), with the visualization help from “Other Utilities” in TomoNet. Following particle re-extraction and one round of “3D auto-refine” under two-binned pixel size, the resulting resolution for the C2 curved SLP hexamer structure is 8.5 Å. The defocus value for each micrograph was initially determined by CTFFIND4 (*53*), and the estimated defocus value was used as input for the “3D auto-refine” and “CTF refinement” jobs in Relion4. Resolution was calculated on the “Remote 3DFSC Processing Server” (*54*) with a mask covering one hexamer, and the global resolution reported above is based on the “gold standard” refinement procedures and the 0.143 Fourier shell correlation criterion. The related data collection parameters are listed in table S6.

### Identification of S-layer– and sheath-associated proteins

An in vivo biotinylation procedure (*30*) was used to label cell surface exposed *M. hungatei* envelope proteins. Harvested cells were suspended in culture medium lacking added primary amines and washed three times. The cell pellet (~1 mg) was resuspended in 5 ml medium, and 5 mg of EZ-Link Sulfo-NHS-LC-LC-Biotin (sulfosuccinimidyl-6-[biotinamido]-6-[hexanamido] hexanoate) was added, and the mixture incubated aerobically at room temperature for 30 min. A second washed cell culture aliquot control (i.e., unlabeled cells) was prepared without label present. Following addition of Streptavidin-coated magnetic beads (200 μl of Dynabeads MyOne Streptavidin T1, Invitrogen, binding capacity ~0.2 μg/μl biotinylated protein), the resulting mixture was incubated at room temperature for 30 min. The beads were washed six times with 200 μl of TPBS/SDS (phosphate-buffered saline/0.1% Tween 20 (v/v)/1% SDS (w/v)] to remove nonspecifically bound proteins. Biotinylated proteins were eluted by heating the beads to 95°C in NuPAGE LDS sample buffer (Invitrogen). After pelleting the beads, the recovered eluate was analyzed by SDS-PAGE following our published procedures (*30*). Sypro Ruby–stained (Invitrogen) protein bands were excised from the gels and analyzed as described (*55*).

To identify *M. hungatei* JF1 sheath-associated proteins, a mild CHAPS detergent cell extraction procedure was used to remove proteins unassociated with the sheath, followed by Enhanced Filter-Aided Sample Preparation (eFASP)-based quantitative mass spectrometry (*56*). Cells (0.5 ml) were suspended in an equal volume of 0.1-mm glass beads and mechanically disrupted using a Biospec Mini-Bead Beater-1 device (Biospec Products, Bartlesville, OK) for 5 min at 5000 rpm at 5°C. The sheath was harvested by microfuge centrifugation for 10 min at 13,000 rpm, and the pellet was re-suspended in 1 ml of 0.1 N NaOH, incubated for 30 min at room temperature and washed twice in 1 ml of TBS [100 mM NaCl and 50 mM tris (pH 7.2)]. It was again centrifuged for 5 min at 3000 rpm. To remove loosely associated cell debris, the pellet fraction was resuspended in TBS buffer containing 2% CHAPS detergent (3-[(3-cholamidopropyl) dimethylammonio]-1- propanesulfonate, Sigma-Aldrich) and incubated 30 min at room temperature and then washed twice with TBS to remove loosely associated cell debris. The resulting pellet was then resuspended in 1 ml TBS and stored at 4°C before quantitative mass spectrometry analysis (*56*). Without CHAPS present, both paralogs were observed in similar amounts in contrast to that shown in Fig. 5B.

### Sequence alignment, modeling, and visualization

Pairwise sequence alignment was performed using the EMBOSS Needle online server (*57*). The predicted models for *M. hungatei* SLP paralogs were either downloaded from the AlphaFold Protein Structure Database (*28*) or generated with AlphaFold2 Google Colab (*58*). IMOD was used to visualize 2D tomographic slices of the reconstructed tomograms and subtomogram averages. The “*v2”* function from EMAN (*59*) was used to show continuous slices of the STA result in fig. S1. The model-to-map cross-correlation coefficient was calculated by Phenix (*60*). UCSF ChimeraX (*61*) was used to generate the supplementary movies and to visualize the tomograms and resulting subtomogram averages, along with the corresponding AlphaFold-predicted atomic models.

## Supplementary Materials

### The PDF file includes:

Figs. S1 to S12 Tables S1 to S6 Legends for movies S1 to S3 References
