# Supplementary material for: Composition and in situ structure of the Methanospirillum hungatei cell envelope and surface layer
Source: Sci Adv. 2024 Dec 13;10(50):eadr8596. doi: 10.1126/sciadv.adr8596 (PMC11641113; doi:10.1126/sciadv.adr8596)
Supplement: Supplementary file 1 — Figs. S1 to S12 Tables S1 to S6 Legends for movies S1 to S3 References [file sciadv.adr8596_sm.pdf]

Science Advances
AAAS

Supplementary Materials for

Composition and in situ structure of the *Methanospirillum hungatei* cell envelope and surface layer

Hui Wang *et al.*

Corresponding author: Robert P. Gunsalus, robg@microbio.ucla.edu; Z. Hong Zhou, hong.zhou@ucla.edu

Sci. Adv. **10**, eadr8596 (2024)
DOI: 10.1126/sciadv.adr8596

The PDF file includes:

Figs. S1 to S12
Tables S1 to S6
Legends for movies S1 to S3
References

Other Supplementary Material for this manuscript includes the following:

Movies S1 to S3

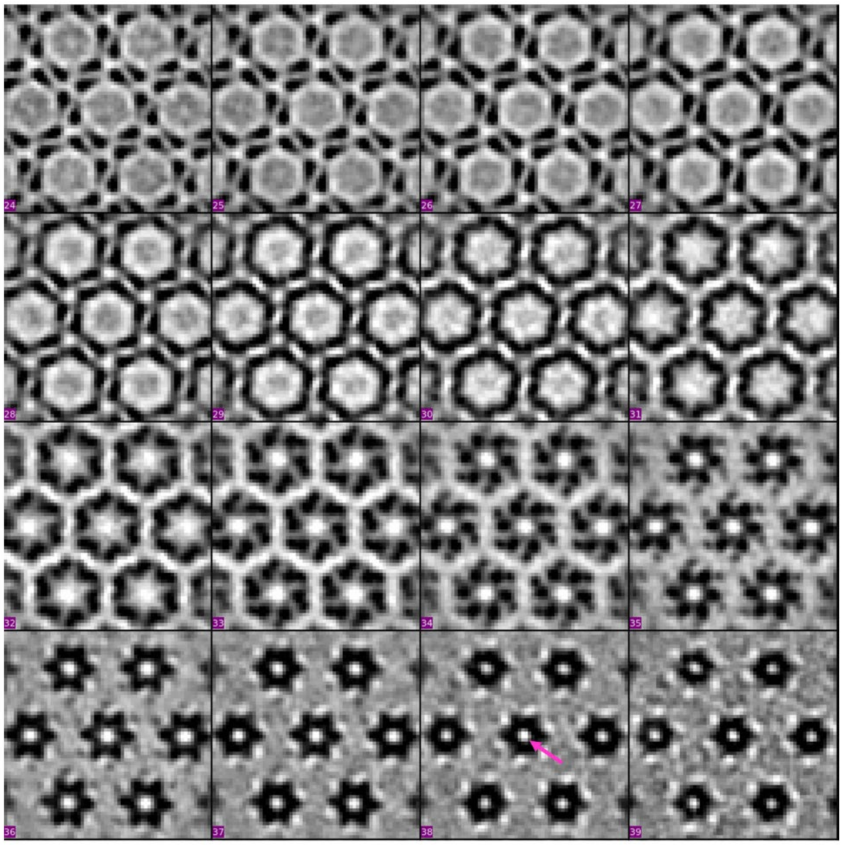

Fig. S1 | Representative slices of the initial STA result.

The initial STA result of the S-layer subunit, without imposing symmetry, suggests it is 6-fold symmetric. The arrow indicated a central pore in the cap region.

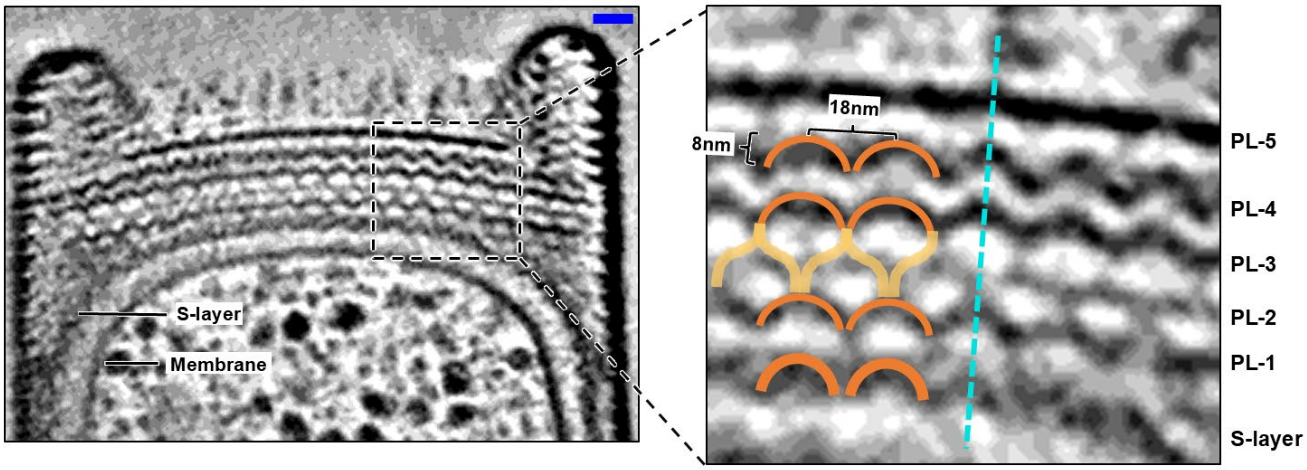

Fig. S2 | *M. hungatei* end plug layers (PLs).

There are 5 layers in the end plug region directly stacked on the S-layer. PL-1, PL-3 and PL-4 have a similar topology as the S-layer, and their cap domains are aligned on the same axis (dashed cyan line). PL-2 has weak density and features a “Y-shaped” repeating subunit that bridges PL-1 and PL-3. PL-5 is the outermost layer and relatively flat. Scale bar, 25 nm.

A

Identity: 770/870 (88.5%), Similarity: 800/870 (92.0%), Gaps: 31/870 (3.6%)

```
Mhun_2513   1 MNARFAVVMMVLVALAIMAFPATAAINKIPAGGEVFLGEKGLDVSAATGGSSQIAWWQPGTNTETEQPADIQQVTNAQSFYVSPDIFVGKTGNWYQWNGS 100
Mhun_2263   1 MNAKFAVVMMVLVALALVAFPASAAINKIPAGGEVFLGEKGLDVSAATGGSSQIAWWQPGTNTETEQPADIQQVTNAQNFYVSPDIFVGKTGNWYQWNGS 100

Mhun_2513 101 VKGPLAFNIKEPSLNLKVWDGSVNEDVTGKAIPVGNYGNFVVETNMQSIITRPGYQPADAPFKIKVKSADGGVYTNLVGNNGKEISLTNLAVNQQLWYWV 200
Mhun_2263 101 VKGPLAFNIKEPSLNLKIWDGSVNEDVTGKAIPVGNYGNFVVETNMQSIITRPGYQPADAPFKIKVKSADGGVYTNLVGNNGKEISLTNLAVNQQLWYWV 200

Mhun_2513 201 SPDEKHTEPASNDGWNTAAEDKYGNRMYKAGTYTVWAECNANKMKDQYKSPDGSDYTGKTISAVKSVQIATDQVKIEANKDTVVRGNPFSVTITGVPNAE 300
Mhun_2263 201 SPDNKHTEPASNDGWNTAAEDKYGNRMYKAGTYTVWAECNANGMKDQYTAPDGSDYTGKTISAVKSVQIATDQVKIEANKDTVVRGNPFSVTITGVPNAE 300

Mhun_2513 301 YYVWAKGSGSMTGQPDDQPPMILSTQADVKQDAAAGPYEIGKYQYEGGAGKSIKQDVPDDPEFHGTKFYALVKLNSSGTRTVEWKTSKDTKDKKYTIRVE 400
Mhun_2263 301 YYVWAKGTGSMTGQPDDQPPMILDAQVDVKQDAAAGPYEIGKYQYEGGAGKSIKQDVPDDPEFHGTKFYALVKLNSSGTRTVEWKTSKDTKDKKYTIRVE 400

Mhun_2513 401 RKSGSQYKSDEVDVKVEKGDVTIVASGDQSYYLGEEVKLSGTNSETDVTYLFITGPNLPNAGGMLKSPRKEVNNDQPASFDQADVQDDDTYEFKWQTANL 500
Mhun_2263 401 RKSGSQYKSDEVDVKVEKGDVTIVASGDQSYYLGEEVKLSGTNSETDVTYLYITGPNLPTRGGMLKSPRKEVNNDQPASFDQADVQDDDTYEFKWQTANL 500

Mhun_2513 501 EMDAGTYTVYAVSAPRDKDHLSDAQYDTVSLVIKKPYIQATASASVVAKGDKMYLRGTAEGDPSKGVAIWILGKNKVLYATESVNDDMSFEHEIKSAETA 600
Mhun_2263 501 EIDAGTYTVYAVSAPRDKDHLSDAQYDTVSLVIKKPYIQATASASVVAKGDKMYLRGTAEGDPSKGVAIWILGKNKVLYATESVNDDMSFEHEIKSATTS 600

Mhun_2513 601 SLYAGQYFVVIQHPMYNNEFDVYPDNPLEPQKILGAYPTRDSEVFRIGGAGALQGTDAAEALVRAIDSAMVDDIYTKMQFLVEEPKVSINPIGEQAVGSK 700
Mhun_2263 601 SLYAGQYFVVVQHPMYNNEFDVYPDNPLEPQYVYGSYPTRNSQVFKLGGSGALQGTDAAEALIQAINSPMVDDTYTKMQFLVEEPKITINPIGEQAVGSK 700

Mhun_2513 701 FEITGTTNLMYDDNDLLVEVTSSSFKPTDKSQSGEFNGATGTIKVQQGTDGLNKWSFTVDASTFKPDEYIVRVSGVTTDVVETALFNVVEAGAAP-VAPV 799
Mhun_2263 701 FEIAGTTNLMYDDNDLLVEVTSSSFKPTDKSQSGEFSGATGTVKVQQGTDGLNKWAFNVDASTFKPDEYIVRVSGVTTNTVETALFNVVEAGAIPTVAPT 800

Mhun_2513 800 VAPTEAPVVEVTAEAPAVEEPVAEETNATETNTTEEAPVAEEPVNKTA---------------------- 847
Mhun_2263 801 VAPTEAPVVN--------ETPTPEPTTVPPTPEPTTVPPTPEPTQQSPGFGALVALAGLGAVAFFVMRRN 862
```

B

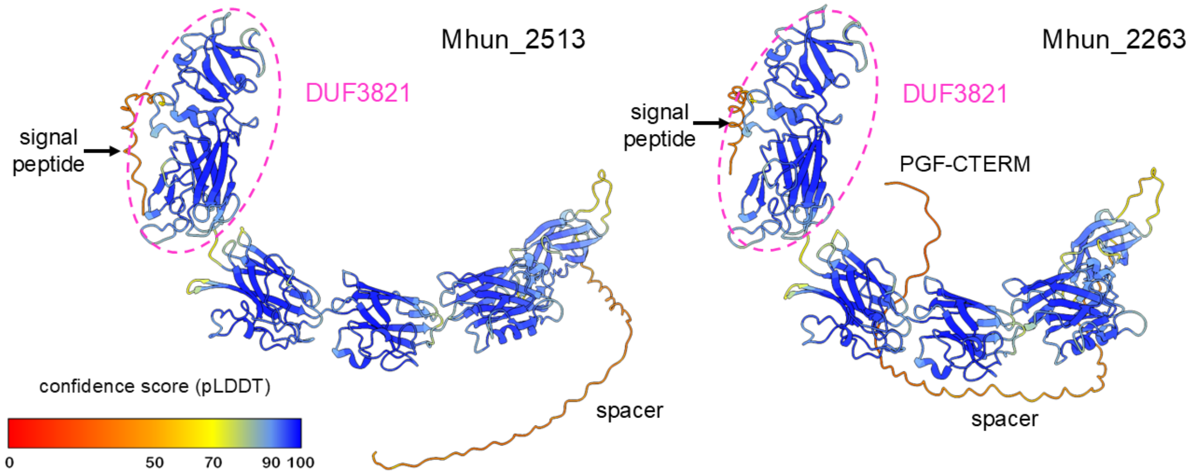

Fig. S3 | Sequence and structural comparison of two major *M. hungatei* SLP paralogs.

(A) Aligned primary sequences of Mhun_2513 and Mhun_2263 proteins indicate high identity and similarity, except that Mhun_2513 is missing the PGF-CTERM domain. Corresponding portions of the N-terminal signal-peptide, DUF3821 and C-terminal PGF-CTERM domains are colored in blue, magenta and red, respectively. **(B)** AlphaFold-predicted models suggest they are also structurally similar, with 6 Ig-like domains. Lower confidence scores for the linker regions suggest that the predicted spatial arrangement of Ig-domains is inaccurate. Magenta boxes enclose the DUF3821 domain.

A

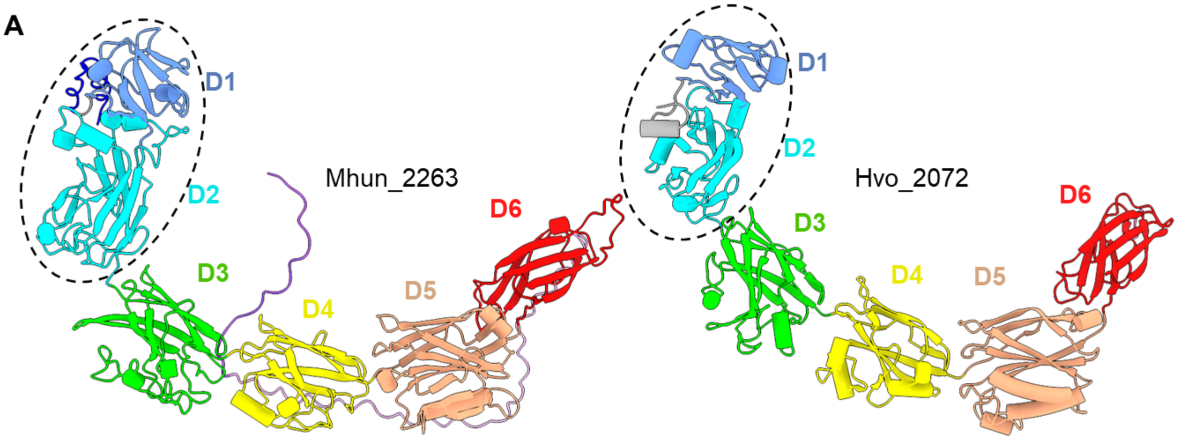

B Identity: 230/995 (23.1%), Similarity: 352/995 (35.4%), Gaps: 301/995 (30.3%)

```
Mhun_2263  ---MN-----AKFAVVMMVLVALALVAFPASAA-------------INKIPAGGEVFLGEKGLDVSAATGGSSQIAWWQPGTNTE--TEQ  67
Hvo_2072   MTKLKDQTRAILLATLMVTSVFAGAIAFTGSAAAERGNLDADSESFNKTIQSGDRVFLGEEIST-DAGLGASNPLLTGTAGNSEGVSLDL  89

Mhun_2263  PADIQQVTNAQNFYVSPDIFVGKTGNWYQWNGS--VKGPLAFNIKEPSLNLKIWDGSVNEDVTGKAIPVGNYGNFVVETNMQSIITRPGY  155
Hvo_2072   SSPIPQTTENQP------------LGTYDVDGSGSATTPN-VTLLAPRITDSEILTSSGGDVTGSAISSSDAGNLYVNADYNYE------  160

Mhun_2263  QPADAPFKIKVKSADGGVYTNLVGNNGKEISLTNLAVNQQLWYWVSPDNKHTEPASNDGWNTAAEDKYGNRMYKAGTYTVWAECNANGMK  245
Hvo_2072   -SAE-KVEVTVEDPSGTDITNEV-----------LSGTD---TFVDDGSIGSTSSTGGGVGI------DMSDQDAGEYTIILEGAE----  224

Mhun_2263  DQYTAPDGSDYTGKTISAVKSVQIATDQVKIEANKDTVVRGNPFSVTITGVPNAEYYVWAKGTGSMTGQPDDQPPMILDAQVDVKQDAAA  335
Hvo_2072   -------DLDFGDA-TETMTLTISSQDEIGIELDSESVTQGTDVQYTVTNGIDGNEHVVAMD-------------LSDLQNDATTEQAK  292

Mhun_2263  GPY-EIGKYQYEGGAGKSIK--QDVPDDPEFH-GTKFYALVKLNSSG---TRTVEWKTSKDTKDKKYTIRVERK-SGSQYKSDEVDVKVE  417
Hvo_2072   EVFRNIGDTSEVGIANSSATNTSGSSTGPTVETADIAYAVVEIDGASAVGGIETQYLDDSEVDLEVYDAGVSATAAVGQDATNDITLTIE  382

Mhun_2263  KGDVTIVASGDQSYYLGEEVKLSGTNSETDVTYLYITGPNL---PTRGGMLKSPRKEVNNDQPASFDQADVQDDDTYEFKWQTANLEIDA  504
Hvo_2072   EGGTT-LSSPTGQYVVGSEVDINGTATSSDSVAIYVRDDGDWQLLEIGG-----DNEISVDSDDTFEEEDIALSG---LSGDGSSILSLT  463

Mhun_2263  GTYTVYAVSAPRD--------KDHLSDAQYDT-----VSLV-----IKKPYIQATASASVVAKGDKMYLRGTAEGDPSKGVAIWILGKNK  576
Hvo_2072   GTYRIGVIDASDADVGGDGSVDDSLTTSEFTSGVSSSNSIRVTDQALTGQFTTINGQVAPV-ETGTVDINGTASGANS-VLVIFVDERGN  551

Mhun_2263  VLYATESVNDDMSFEHEIKSATTSSLYAGQYFVVVQHPMYNNEFDVYPDNPLEPQYVYGSYPTRNS---QVFKLGGS-GAL-QGTDAAEA  661
Hvo_2072   VNYQEVSVDSDGTYDEDDIT---VGLTQGRVTA---HI-----LSVGRDSA----IGDGSLPSGPSNGATLNDLTGYLDTLDQNNNNGEQ  626

Mhun_2263  LIQAINSPMVDD-----TYTKMQFLVEEPKITI----------NPIGEQAVGSKFEIAGTTNLMYDDNDLLVEVTSSSFKPTDKSQSGEF  736
Hvo_2072   INELIASETVDETASDDLIVTETFRLAESSTSIDSIYPDAAEAAGINPVATGETMVIAGSTNLKPDDNTISIEVTNEDGTSVA-------  709

Mhun_2263  SGATGTVKVQQGTDGLNKWAFNVDASTFKPDEYIVRVSGV-TTNTVETALFNVVEAGAIPTVAPTVAPTEAPVVNETPTPEPTTVPPTPE  825
Hvo_2072   ------LEDTDEWNNDGQWMVEIDTTDFETGTFTVEADDGDNTDTVNVEVVSEREDT---TT-SSDNATDTTTTTDGPTETTTT--AEPT  787

Mhun_2263  PTTVPPT--PEPTQQSPGFGALVALAGLGAVAFFVMRRN-   862
Hvo_2072   ETTEEPTEETTTSSNTPGFGIAVALVALVGAALLALRREN   827
```

D1 D2 D3 D4 D5 D6
Signal-peptide spacer PGF-CTERM

Fig. S4 | Structural and sequence comparison of *M. hungatei* and *H. volcanii* SLPs.

(A) Left: AlphaFold-predicted monomer model of Mhun_2263. Right: Atomic model of Hvo_2072 monomer (PDB: 7ptr). All the Ig-like domains are aligned nearly identically, despite varying lengths and relatively low similarity. D1 and D2 of Mhun_2263, together corresponding to DUF3821 in *M. hungatei*, are notably larger than those of Hvo_2072. **(B)** Aligned primary sequences of Mhun_2263 and Hvo_2072, with their signal-peptide elements (blue), D1-D6 Ig-like domains (light blue, cyan, green, yellow, tan and red), spacer (pink) and PGF-CTERM (purple) highlighted in color underneath.

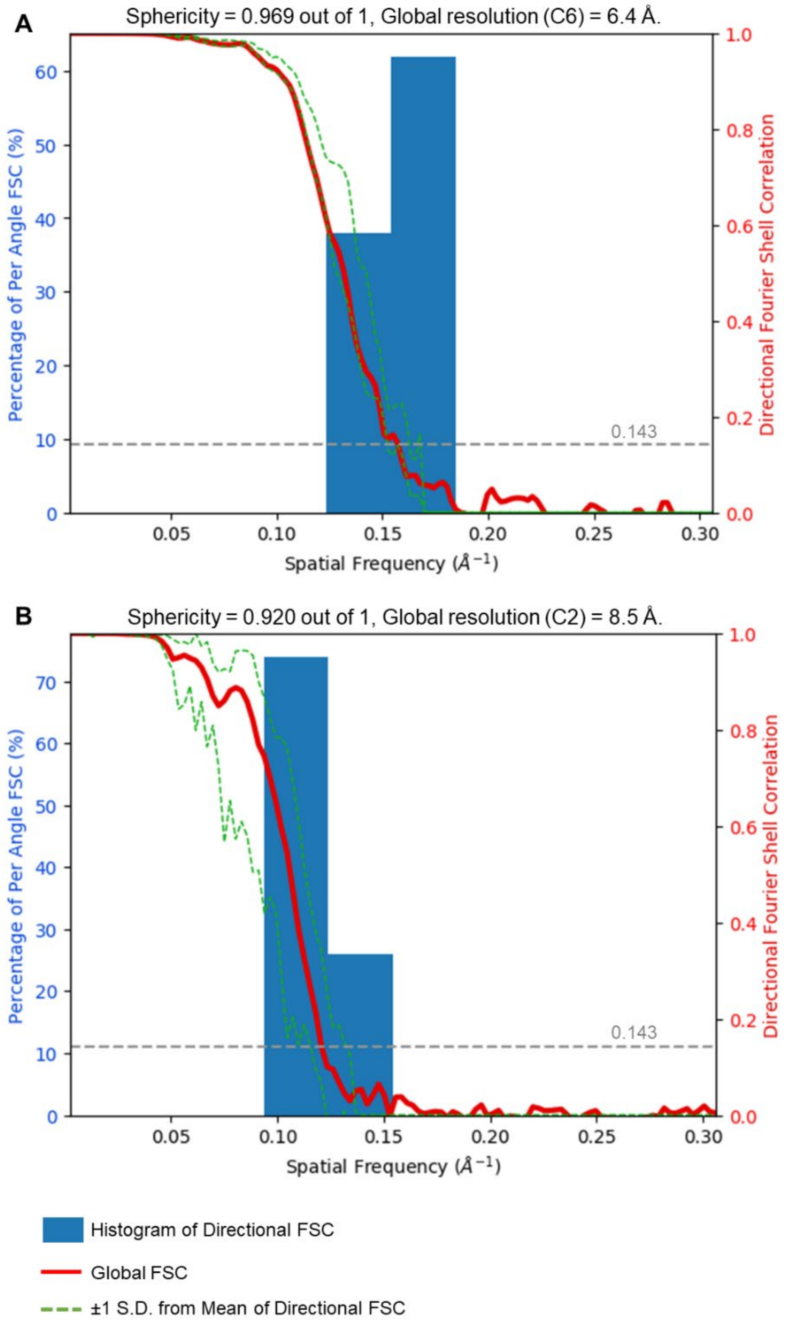

Fig. S5 | Resolution of STA results.

(A-B) Directional Fourier shell correlation (FSC) curves of the C6 and C2 STA reconstructions, indicate global resolutions of 6.4 Å and 8.5 Å, respectively.

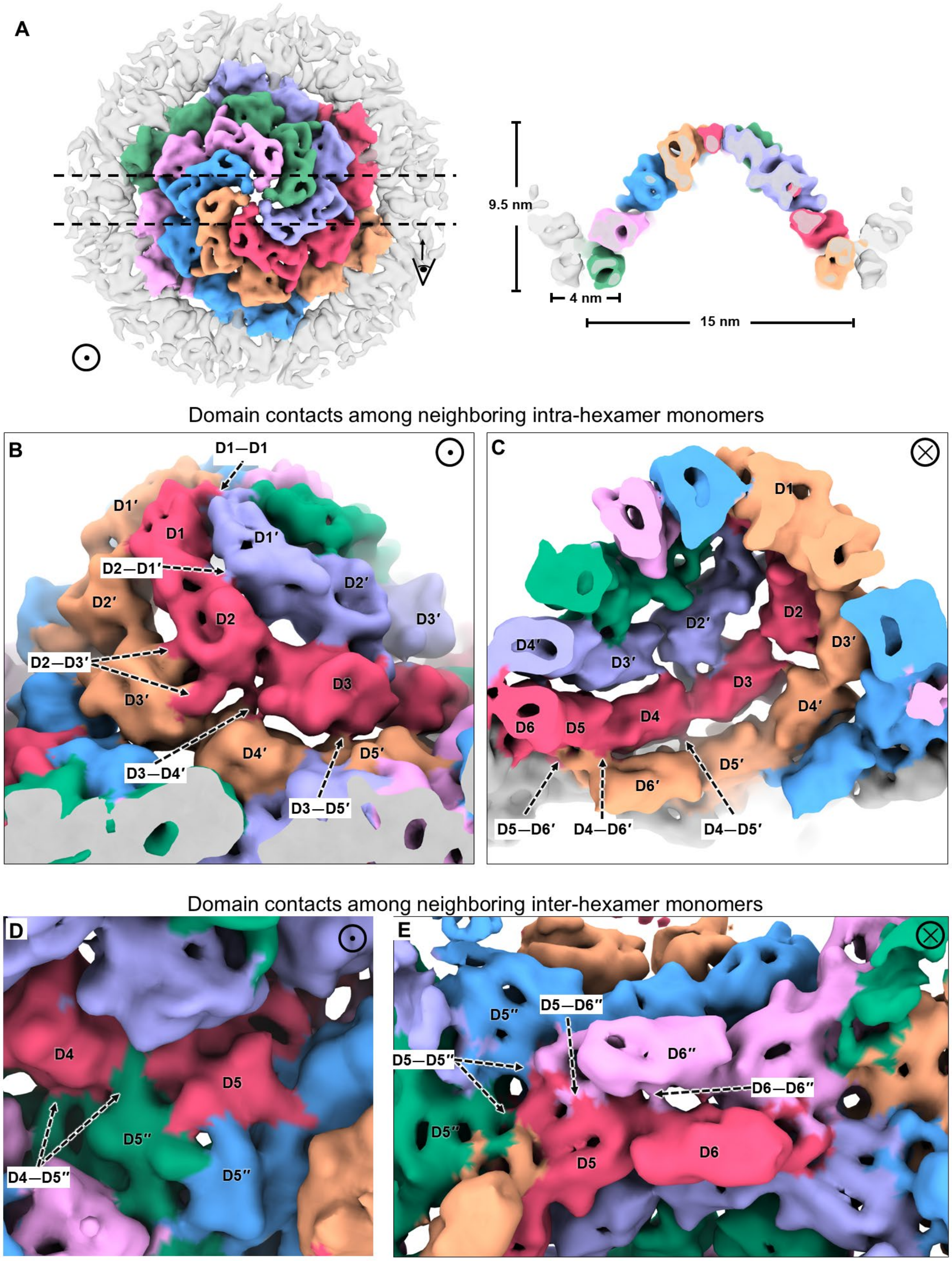

Fig. S6 | Domain interactions among SLP monomers.

(A) Two orthogonal views of the segmentation map of the SLP hexamer, with each monomer colored differently, showing the overall dimensions and intensive inter-monomer interactions. (**B-E**) Domain interactions between neighboring SLP monomers within a hexamer and between hexamers. Dashed arrows point to the linking densities. SLP monomers were colored differently in the STA map.

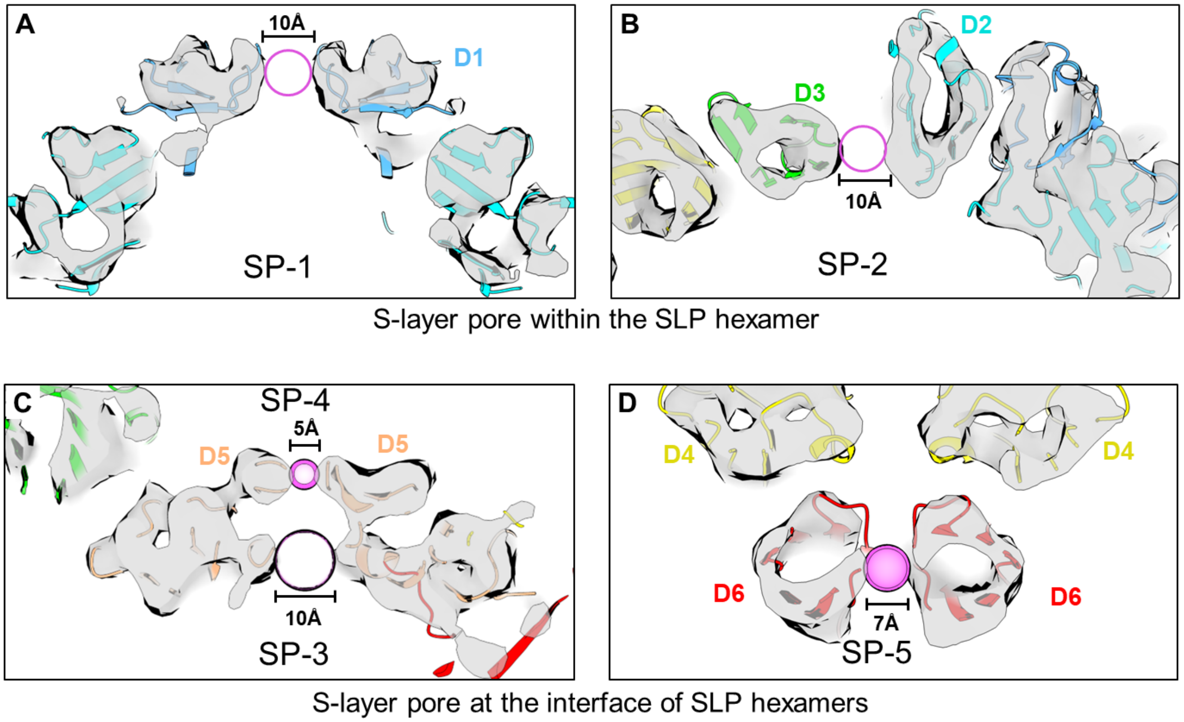

Fig. S7 | Cross sectional views of the *M. hungatei* S-layer pores (SPs).

(A-D) SP-1, -2, -3, -4 and -5 are located either within the SLP hexamer or at the interface between SLP hexamers. Pink spheres of varying sizes were placed at the center of each pore to indicate their size.

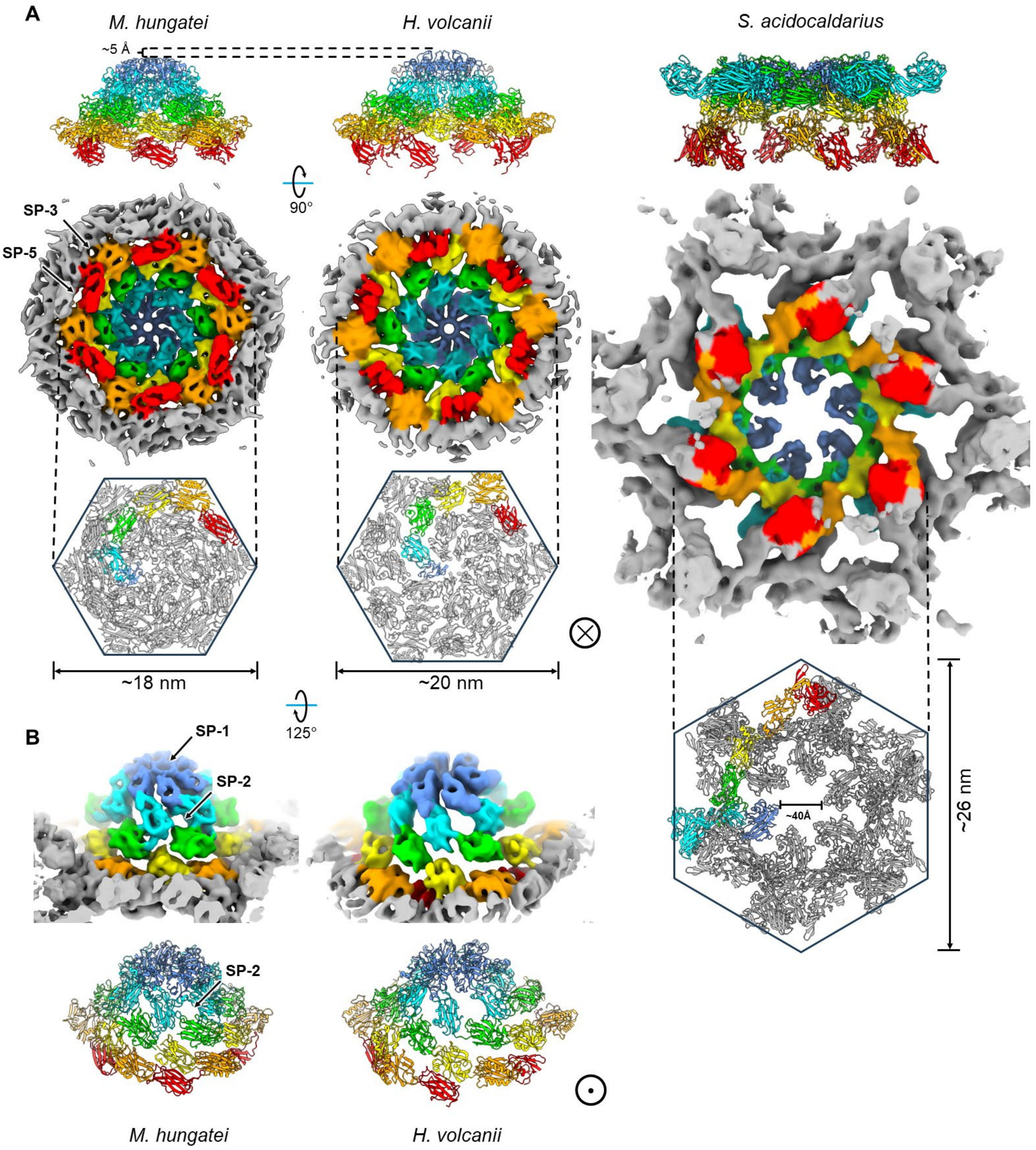

Fig. S8 | Differences in shape and dimension of SLP hexamers among different species.

(A) Comparison of SLP hexamer structures among archaeal species (*M. hungatei, H. volcanii* and *S. acidocaldarius*) with Ig-like domains colored differently, illustrating differences in their shapes and dimensions. **(B)** Comparison showing the SLP hexamer is more compactly assembled in *M. hungatei* compared to *H. volcanii*.

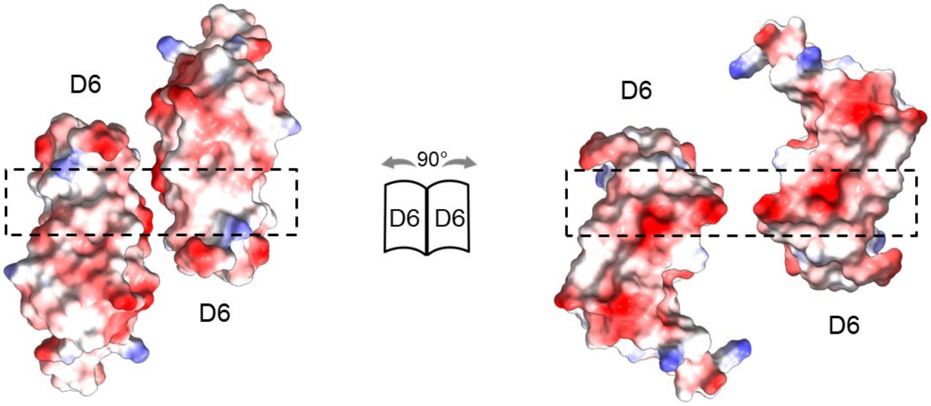

Fig. S9 | Charge distribution of opposing D6 domains at the dimeric inter-hexamer interface. An open-book view showing two negatively charged surfaces of opposing D6 domains.

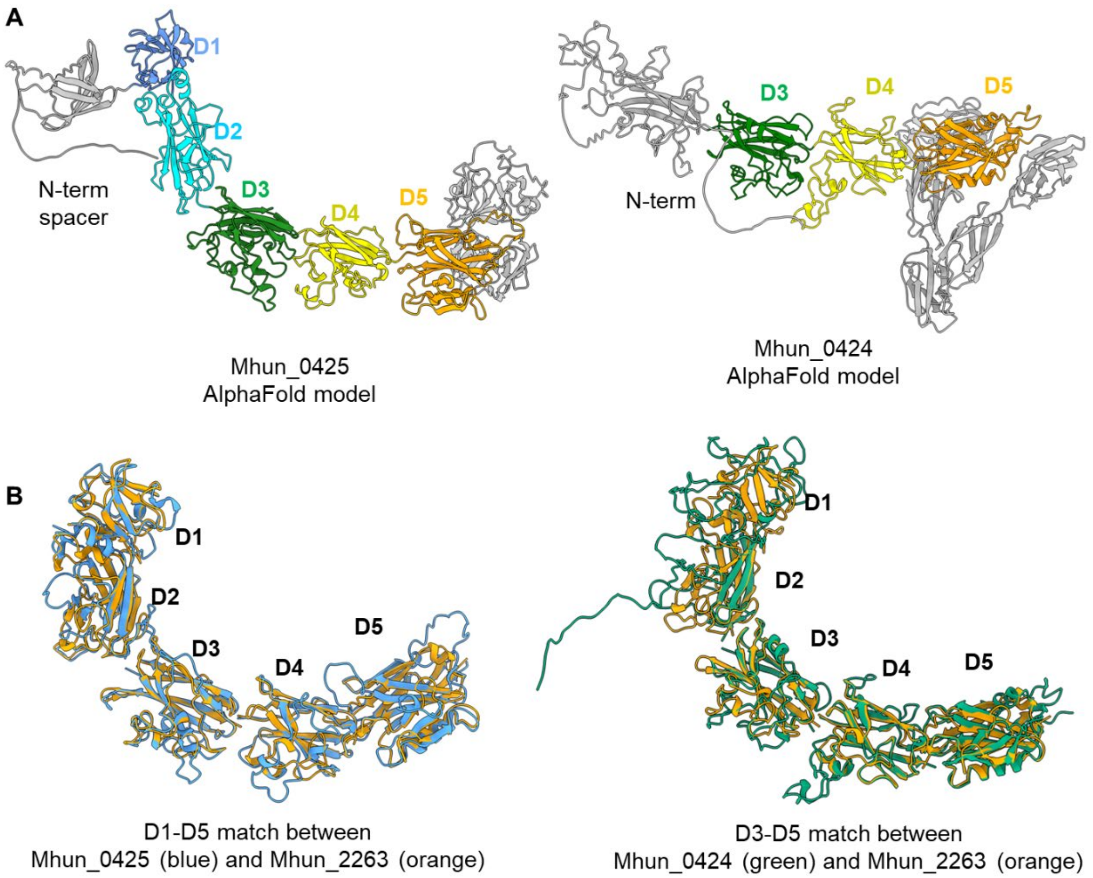

Fig. S10 | AlphaFold model of SLP paralogs Mhun_0425 and Mhun_0424.

(A) AlphaFold models of Mhun_0425 and Mhun_0424 showing their extended N-terminal domains. **(B)** Ig-like domains demonstrating structural conservation in Mhun_0425 (D1-D5) and Mhun_0424 (D3-D5) compared to Mhun_2263.

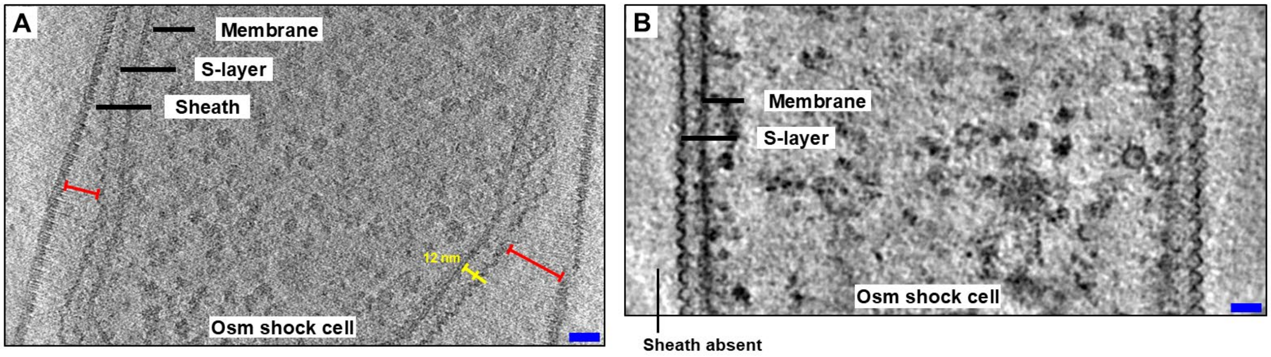

Fig. S11 | Osmatic shock detaches the sheath layer but retains the S-layer.

(A) An osmotically shocked *M. hungatei* cell showing regional delamination of the sheath layer (i.e., the uniform distance is not maintained) and the S-layer remains attached to the cell membrane. **(B)** An osmotically shocked *M. hungatei* cell showing complete detachment of its sheath layer. Scale bars, 25 nm.

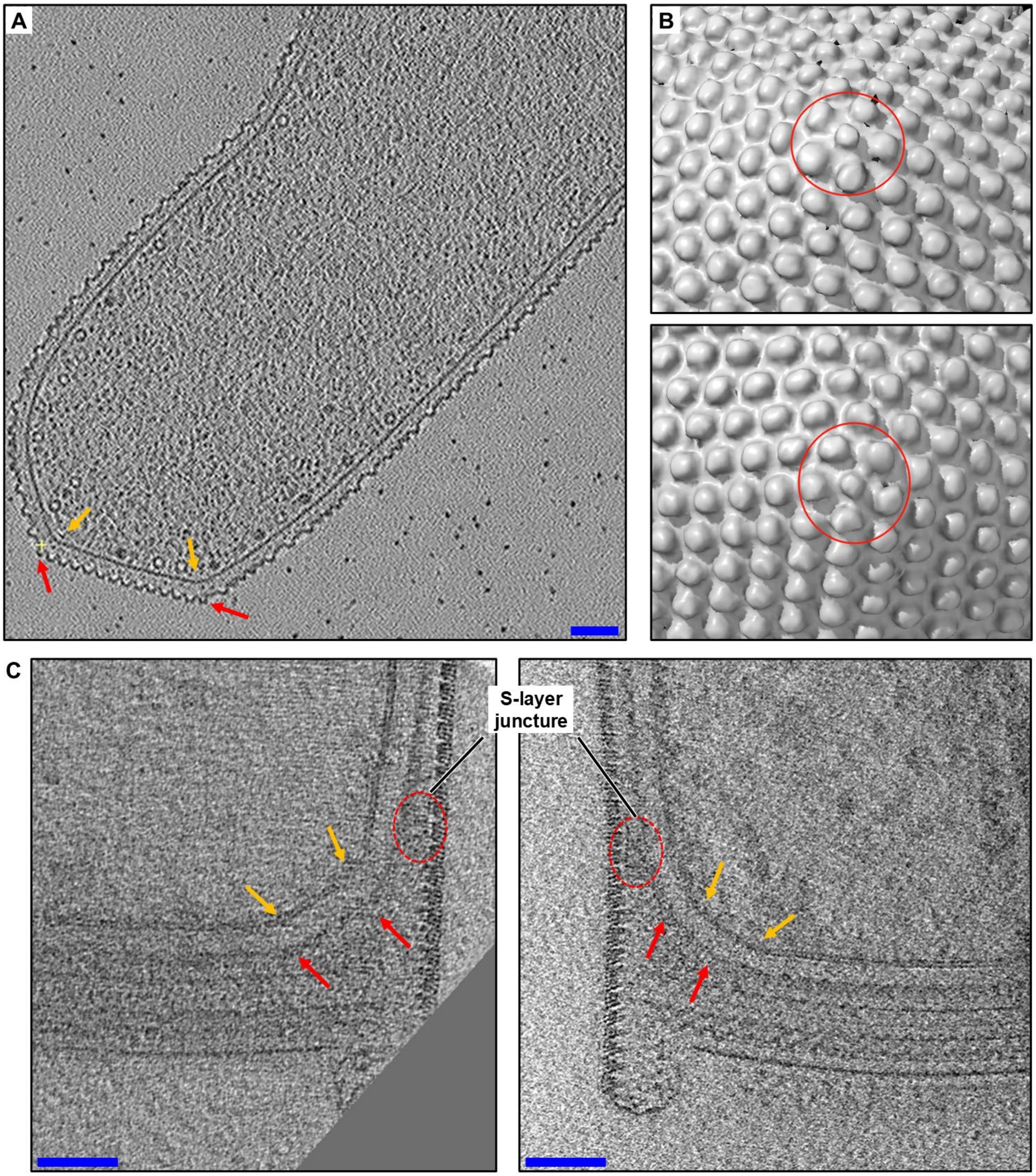

Fig. S12 | Pentamers observed in the assembly of the *Methanoregula formicica* S-layer lattice.

(A) A slicer view of an *M. formicica* cell showing the outermost S-layer. **(B)** Pentamers are observed in *M. formicica* cellular tomograms (circled), contributing to the vertices and the sharp edges on the S-layer lattice and cell membrane (indicated by arrows in panel (**A**)). **(C)** Sharp edges of S-layer lattice and cell membrane are also observed in the *M. hungatei* end plug region (indicated by arrows). Scale bars, 50 nm.

Table S1 | Properties of the surface layer proteins of *M. hungatei JF1* and *H. volcanii*.

Property	Mhun_2513	Mhun_2263	Hvo_2072
Predicted size (Da)	91,736	93,246	85,188
Amino acids (AA)	847 AA	862 AA	827 AA
Amino acids w/o SP	823	838	793
Annotation	hypothetical	hypothetical	cell surface glycoprotein
Observed size	~116 kDa	~116 kDa	~150 kDa (*62*)
Signal-peptide	24 AA predicted	24 AA predicted	34 AA
DUF3821 (pfam/InterPro)	DUF3821	DUF3821	none
PGF-CTERM	absent	yes	yes
Predicted size after cleavage (Da)	89,215	90,799	82,526 (~8 kDa smaller)
Isoelectric point (pI)	4.68	4.57	3.37
N- or O-linked glycan	unknown	unknown	N-linked

Table S2 | Comparison of Ig-like and spacer domains between *M. hungatei* and *H. volcanii* SLP.

Domain	Mhun_2263 (AA)	Hvo_2072 (AA)	Domain size difference (AA)
D1	84	73	+11
D2	158	119	+39
D3	146	140	+6
D4	117	124	-7
D5	148	146	+2
D6	105	100	+5
spacer	50	49	+1

Table S3 | The plasticity of the hexamer tile as measured by the RMSD between atom pairs in D1-D6 domains between flat and curved conformations shown in Movie S3. The asterisk (*) denotes the calculations were performed only on the four D6 domains with obvious movements.

Domain in one SLP tile	RMSD between C6 and C2 (Å)
D1 (6 copies)	RMSD between 3792 atom pairs is 4.9
D2 (6 copies)	RMSD between 7314 atom pairs is 2.6
D3 (6 copies)	RMSD between 6834 atom pairs is 2.8
D4 (6 copies)	RMSD between 5406 atom pairs is 3.2
D5 (6 copies)	RMSD between 6966 atom pairs is 3.5
D6 (6 copies)	RMSD between 4704 atom pairs is 3.8
D6 (4 copies) *	RMSD between 3136 atom pairs is 4.4

Table S4 | The *M. hungatei* JF1 SLP paralogs.

Locus tag *1	SP/TM *1	DUF3821 *2	# Ig-like domains *5	Size (Da)	other domains
Mhun_0799	no/yes	no	1	21,655	
Mhun_1876	no/yes	yes	2	33,341	
Mhun_0651	yes/no	yes	2	36,506	
Mhun_0459	no/no	yes	2	37,861	
Mhun_3033	no/yes	yes	2	38,583	
Mhun_0945	no/yes	no	3	42,699	2x PKD_4 *3
Mhun_1948	yes/no	yes	5	66,876	
Mhun_2513	yes/yes	yes	6	91,736	
Mhun_2263	yes/yes	yes	6	93,246	PGF-CTERM *4
Mhun_2759	no/yes	yes	6	97,029	
Mhun_0422	no/yes	no	7	105,329	4x PKD_4
Mhun_0423	no/yes	yes	8	120,038	3x PKD_4
Mhun_0424	yes/yes	yes	10	141,075	4x PKD_4
Mhun_0425	yes/yes	yes	10	135,229	2x PKD_4

*1 Data from the IMG-JGI (https://img.jgi.doe.gov); SP, signal-peptide; TM, transmembrane element

*2 DUF3821 = pfam12863 = IPRO24277

*3 PKD_4 = pfam18911 = PKD domain = IPR000601

*4 PGF-CTERM = pfam18204 = no IPRO #

*5 Measurement based on AlphaFold prediction

Table S5 | RMSD of atom pairs in D1-D5 domains between Mhun_2263 and two paralogs, Mhun_0425 and Mhun_0424.

Domains	RMSD: Mhun2263 vs Mhun0425 (Å)	RMSD: Mhun2263 vs Mhun0424 (Å)
D1	**0.62** among 66 pruned atom pairs<br>2.25 among all 83 atom pairs	N/A
D2	**0.96** among 79 pruned atom pairs<br>6.21 among all 149 atom pairs	N/A
D3	**0.75** among 111 pruned atom pairs<br>3.69 among all 146 atom pairs	**0.77** among 83 pruned atom pairs<br>4.80 among all 141 atom pairs
D4	**0.68** among 75 pruned atom pairs<br>5.82 among all 116 atom pairs	**0.74** among 78 pruned atom pairs<br>3.69 among all 110 atom pairs
D5	**1.08** among 106 pruned atom pairs<br>4.03 among all 143 atom pairs	**1.04** among 83 pruned atom pairs<br>8.58 among all 144 atom pairs

Table S6 | CryoET data collection and processing statistics.

**Data collection**	
Microscope	Titan Krios
Voltage (kV)	300
Total electron exposure (e^{-}/Å^2)	110
Slit width (eV)	20
Detector	K2
Defocus range (μm)	-1.5 to -4.0
Pixel size (Å)	1.63
Software	SerialEM
Tilt-series range	±60°
Tilt-series increment	±3°
Tilt-series scheme	dose symmetry
Tilt-series used	36
**Data processing**	
Software: tilt-series alignment	IMOD
Software: particle picking	TomoNet
Software: final reconstruction	Relion4
Initial particle images	64,093
Pixel size final reconstruction (Å)	1.63
Final particle images	46,550; 27,653
Final Box-size (px)	288; 228
Symmetry imposed	C6; C2
Map resolution (Å)	6.4; 8.5
FSC threshold	0.143
EMDB accession codes	EMD-45366; EMD-45367

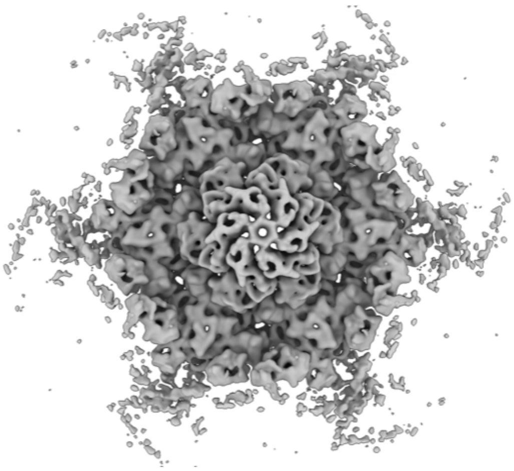

[movie still]

Movie S1 | STA result of SLP hexamer, docked with AlphaFold model of an SLP monomer.

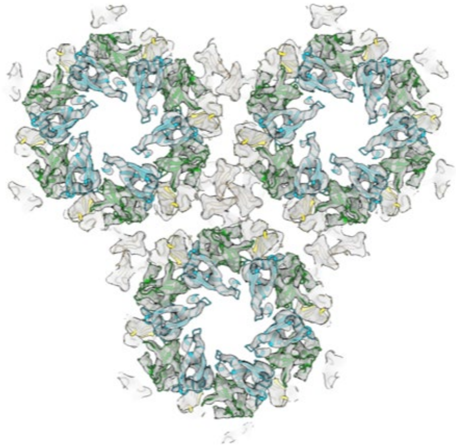

[movie still]

Movie S2 | Slicer views of three connected SLP hexamers at different Z heights, showing the inter-hexamer interfaces and S-layer pores at various locations.

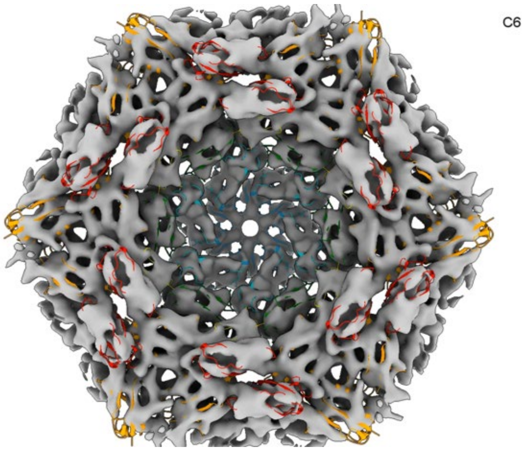

[movie still]

Movie S3 | Morph between flat (C6) and curved (C2) SLP hexamer structures, with corresponding docked AlphaFold models.

REFERENCES AND NOTES

1. R. A. Makula, M. E. Singer, Ether-containing lipids of methanogenic bacteria. *Biochem. Biophys. Res. Commun.* **82**, 716–722 (1978).

2. O. Kandler, H. König, Chemical composition of the peptidoglycan-free cell walls of methanogenic bacteria. *Arch. Microbiol.* **118**, 141–152 (1978).

3. Y. Koga, H. Morii, Biosynthesis of ether-type polar lipids in archaea and evolutionary considerations. *Microbiol. Mol. Biol. Rev.* **71**, 97–120 (2007).

4. J. G. Ferry, P. H. Smith, R. S. Wolfe, *Methanospirillum,* a new genus of methanogenic bacteria, and characterization of *Methanospirillum hungatii* sp.nov. *Int. J. Syst. Evol. Microbiol.* **24**, 465–469 (1974).

5. D. B. Toso, A. M. Henstra, R. P. Gunsalus, Z. H. Zhou, Structural, mass and elemental analyses of storage granules in methanogenic archaeal cells. *Environ. Microbiol.* **13**, 2587–2599 (2011).

6. J. G. Zeikus, V. G. Bowen, Fine structure of Methanospirillum hungatii. *J. Bacteriol.* **121**, 373–380 (1975).

7. A. M. Glauert, The fine structure of bacteria. *Br. Med. Bull.* **18**, 245–250 (1962).

8. M. Sára, U. B. Sleytr, S-layer proteins. *J. Bacteriol.* **182**, 859–868 (2000).

9. S.-V. Albers, B. H. Meyer, The archaeal cell envelope. *Nat. Rev. Microbiol.* **9**, 414–426 (2011).

10. R. P. Fagan, N. F. Fairweather, Biogenesis and functions of bacterial S-layers. *Nat. Rev. Microbiol.* **12**, 211–222 (2014).

11. C. Zhu, G. Guo, Q. Ma, F. Zhang, F. Ma, J. Liu, D. Xiao, X. Yang, M. Sun, Diversity in S-layers. *Prog. Biophys. Mol. Biol.* **123**, 1–15 (2017).

12. G. B. Patel, L. A. Roth, G. D. Sprott, Factors influencing filament length of *Methanospirillum hungatii*. *Microbiology* **112**, 411–415 (1979).

13. U. B. Sleytr, B. Schuster, E.-M. Egelseer, D. Pum, S-layers: Principles and applications. *FEMS Microbiol. Rev.* **38**, 823–864 (2014).

14. A. L. Houwink, A macromolecular mono-layer in the cell wall of Spirillum spec. *Biochim. Biophys. Acta* **10**, 360–366 (1953).

15. A. Lupas, H. Engelhardt, J. Peters, U. Santarius, S. Volker, W. Baumeister, Domain structure of the Acetogenium kivui surface layer revealed by electron crystallography and sequence analysis. *J. Bacteriol.* **176**, 1224–1233 (1994).

16. J. Smit, H. Engelhardt, S. Volker, S. H. Smith, W. Baumeister, The S-layer of Caulobacter crescentus: Three-dimensional image reconstruction and structure analysis by electron microscopy. *J. Bacteriol.* **174**, 6527–6538 (1992).

17. M. Sumper, E. Berg, R. Mengele, I. Strobel, Primary structure and glycosylation of the S-layer protein of Haloferax volcanii. *J. Bacteriol.* **172**, 7111–7118 (1990).

18. M. Firtel, G. Southam, G. Harauz, T. J. Beveridge, Characterization of the cell wall of the sheathed methanogen Methanospirillum hungatei GP1 as an S layer. *J. Bacteriol.* **175**, 7550–7560 (1993).

19. E. Baranova, R. Fronzes, A. Garcia-Pino, N. Van Gerven, D. Papapostolou, G. Péhau-Arnaudet, E. Pardon, J. Steyaert, S. Howorka, H. Remaut, SbsB structure and lattice reconstruction unveil Ca^{2+} triggered S-layer assembly. *Nature* **487**, 119–122 (2012).

20. M. A. Arbing, S. Chan, A. Shin, T. Phan, C. J. Ahn, L. Rohlin, R. P. Gunsalus, Structure of the surface layer of the methanogenic archaean *Methanosarcina acetivorans*. *Proc. Natl. Acad. Sci.* **109**, 11812–11817 (2012).

21. A. von Kügelgen, H. Tang, G. G. Hardy, D. Kureisaite-Ciziene, Y. V. Brun, P. J. Stansfeld, C. V. Robinson, T. A. M. Bharat, *In situ* structure of an intact lipopolysaccharide-bound bacterial surface layer. *Cell* **180**, 348–358.e15 (2020).

22. T. A. M. Bharat, D. Kureisaite-Ciziene, G. G. Hardy, E. W. Yu, J. M. Devant, W. J. H. Hagen, Y. V. Brun, J. A. G. Briggs, J. Löwe, Structure of the hexagonal surface layer on *Caulobacter crescentus* cells. *Nat. Microbiol.* **2**, 17059 (2017).

23. A. von Kügelgen, V. Alva, T. A. M. Bharat, Complete atomic structure of a native archaeal cell surface. *Cell Rep.* **37**, 110052 (2021).

24. A. von Kügelgen, S. van Dorst, K. Yamashita, D. L. Sexton, E. I. Tocheva, G. Murshudov, V. Alva, T. A. M. Bharat, Interdigitated immunoglobulin arrays form the hyperstable surface layer of the extremophilic bacterium *Deinococcus radiodurans*. *Proc. Natl. Acad. Sci.* **120**, e2215808120 (2023).

25. L. Gambelli, M. McLaren, R. Conners, K. Sanders, M. C. Gaines, L. Clark, V. A. M. Gold, D. Kattnig, M. Sikora, C. Hanus, M. N. Isupov, B. Daum, Structure of the two-component S-layer of the archaeon *Sulfolobus acidocaldarius*. *eLife* **13**, e84617 (2024).

26. A. von Kügelgen, C. K. Cassidy, S. van Dorst, L. L. Pagani, C. Batters, Z. Ford, J. Löwe, V. Alva, P. J. Stansfeld, T. A. M. Bharat, Membraneless channels sieve cations in ammonia-oxidizing marine archaea. *Nature* **630**, 230–236 (2024).

27. J. Jumper, R. Evans, A. Pritzel, T. Green, M. Figurnov, O. Ronneberger, K. Tunyasuvunakool, R. Bates, A. Žídek, A. Potapenko, A. Bridgland, C. Meyer, S. A. A. Kohl, A. J. Ballard, A. Cowie, B. Romera-Paredes, S. Nikolov, R. Jain, J. Adler, T. Back, S. Petersen, D. Reiman, E. Clancy, M. Zielinski, M. Steinegger, M. Pacholska, T. Berghammer, S. Bodenstein, D. Silver, O. Vinyals, A. W. Senior, K. Kavukcuoglu, P. Kohli, D. Hassabis, Highly accurate protein structure prediction with AlphaFold. *Nature* **596**, 583–589 (2021).

28. M. Varadi, S. Anyango, M. Deshpande, S. Nair, C. Natassia, G. Yordanova, D. Yuan, O. Stroe, G. Wood, A. Laydon, A. Žídek, T. Green, K. Tunyasuvunakool, S. Petersen, J. Jumper, E. Clancy, R. Green, A. Vora, M. Lutfi, M. Figurnov, A. Cowie, N. Hobbs, P. Kohli, G. Kleywegt, E. Birney, D. Hassabis, S. Velankar, AlphaFold protein structure database: Massively expanding the structural coverage of protein-sequence space with high-accuracy models. *Nucleic Acids Res.* **50**, D439–D444 (2022).

29. H. Wang, J. Zhang, D. Toso, S. Liao, F. Sedighian, R. Gunsalus, Z. H. Zhou, Hierarchical organization and assembly of the archaeal cell sheath from an amyloid-like protein. *Nat. Commun.* **14**, 6720 (2023).

30. D. R. Francoleon, P. Boontheung, Y. Yang, U. Kin, A. J. Ytterberg, P. A. Denny, P. C. Denny, J. A. Loo, R. P. Gunsalus, R. R. Loo, S-layer, surface-accessible, and concanavalin A binding proteins of *Methanosarcina acetivorans* and *Methanosarcina mazei*. *J. Proteome Res.* **8**, 1972–1982 (2009).

31. N. Poweleit, P. Ge, H. H. Nguyen, R. R. O. Loo, R. P. Gunsalus, Z. H. Zhou, CryoEM structure of the *Methanospirillum hungatei* archaellum reveals structural features distinct from the bacterial flagellum and type IV pilus. *Nat. Microbiol.* **2**, 16222 2017.

32. Y. Liu, in *Handbook of Hydrocarbon and Lipid Microbiology*, K. N. Timmis, Ed. (Springer Berlin Heidelberg, 2010), pp. 583–593.

33. D. H. Haft, S. H. Payne, J. D. Selengut, Archaeosortases and exosortases are widely distributed systems linking membrane transit with posttranslational modification. *J. Bacteriol.* **194**, 36–48 (2012).

34. M. F. Abdul Halim, K. R. Karch, Y. Zhou, D. H. Haft, B. A. Garcia, M. Pohlschroder, Permuting the PGF signature motif blocks both archaeosortase-dependent C-terminal cleavage and prenyl lipid attachment for the *Haloferax volcanii* S-layer glycoprotein. *J. Bacteriol.* **198**, 808–815 (2015).

35. H. S. Vollan, T. Tannæs, G. Vriend, G. Bukholm, *In silico* structure and sequence analysis of bacterial porins and specific diffusion channels for hydrophilic molecules: Conservation, multimericity and multifunctionality. *Int. J. Mol. Sci.* **17**, 599 (2016).

36. S. Cohen, M. Shilo, M. Kessel, Nature of the salt dependence of the envelope of a Dead Sea archaebacterium, *Haloferax volcanii*. *Arch. Microbiol.* **156**, 198–203 (1991).

37. E. Johnston, B. Isbilir, V. Alva, T. A. M. Bharat, J. P. K. Doye, Punctuated and continuous structural diversity of S-layers across the prokaryotic tree of life. bioRxiv 2024.05.28.596244 [Preprint] (2024). https://doi.org/10.1101/2024.05.28.596244.

38. I. Bárcena-Uribarri, M. Thein, M. Barbot, E. Sans-Serramitjana, M. Bonde, R. Mentele, F. Lottspeich, S. Bergström, R. Benz, Study of the protein complex, pore diameter, and pore-forming activity of the *Borrelia burgdorferi* P13 porin. *J. Biol. Chem.* **289**, 18614–18624 (2014).

39. G. Southam, M. L. Kalmokoff, K. F. Jarrell, S. F. Koval, T. J. Beveridge, Isolation, characterization, and cellular insertion of the flagella from two strains of the archaebacterium Methanospirillum hungatei. *J. Bacteriol.* **172**, 3221–3228 (1990).

40. T. J. Beveridge, G. D. Sprott, P. Whippey, Ultrastructure, inferred porosity, and gram-staining character of Methanospirillum hungatei filament termini describe a unique cell permeability for this archaeobacterium. *J. Bacteriol.* **173**, 130–140 (1991).

41. R. P. Gunsalus, L. E. Cook, B. Crable, L. Rohlin, E. McDonald, H. Mouttaki, J. R. Sieber, N. Poweleit, H. Zhou, A. L. Lapidus, H. E. Daligault, M. Land, P. Gilna, N. Ivanova, N. Kyrpides, D. E. Culley, M. J. McInerney, Complete genome sequence of *Methanospirillum hungatei* type strain JF1. *Stand Genomic Sci.* **11**, 2 (2016).

42. A. Briegel, D. R. Ortega, A. N. Huang, C. M. Oikonomou, R. P. Gunsalus, G. J. Jensen, Structural conservation of chemotaxis machinery across Archaea and Bacteria. *Environ. Microbiol. Rep.* **7**, 414–419 (2015).

43. Z. H. Zhou, M. Dougherty, J. Jakana, J. He, F. J. Rixon, W. Chiu, Seeing the herpesvirus capsid at 8.5 Å. *Science* **288**, 877–880 (2000).

44. O. Pornillos, B. K. Ganser-Pornillos, M. Yeager, Atomic-level modelling of the HIV capsid. *Nature* **469**, 424–427 (2011).

45. A. Briegel, *Methanoregula formicica* [dataset]. CaltechDATA (2014); https://doi.org/10.22002/tk2zs-xac05.

46. D. N. Mastronarde, Automated electron microscope tomography using robust prediction of specimen movements. *J. Struct. Biol.* **152**, 36–51 (2005).

47. H. Wang, S. Liao, X. Yu, J. Zhang, Z. H. Zhou, TomoNet: A streamlined cryoET software pipeline with automatic particle picking on flexible lattices. bioRxiv 580557 [Preprint] 580557 (2024). https://doi.org/10.1101/2024.02.17.580557.

48. J. Zivanov, J. Otón, Z. Ke, A. von Kügelgen, E. Pyle, K. Qu, D. Morado, D. Castaño-Díez, G. Zanetti, T. A. M. Bharat, J. A. G. Briggs, S. H. W. Scheres, A Bayesian approach to single-particle electron cryo-tomography in RELION-4.0. *eLife* **11**, e83724 (2022).

49. S. Q. Zheng, E. Palovcak, J.-P. Armache, K. A. Verba, Y. Cheng, D. A. Agard, MotionCor2: Anisotropic correction of beam-induced motion for improved cryo-electron microscopy. *Nat. Methods* **14**, 331–332 (2017).

50. J. R. Kremer, D. N. Mastronarde, J. R. McIntosh, Computer visualization of three-dimensional image data using IMOD. *J. Struct. Biol.* **116**, 71–76 (1996).

51. Y.-T. Liu, H. Zhang, H. Wang, C.-L. Tao, G.-Q. Bi, Z. H. Zhou, Isotropic reconstruction for electron tomography with deep learning. *Nat. Commun.* **13**, 6482 (2022).

52. D. Nicastro, C. Schwartz, J. Pierson, R. Gaudette, M. E. Porter, J. R. McIntosh, The molecular architecture of axonemes revealed by cryoelectron tomography. *Science* **313**, 944–948 (2006).

53. A. Rohou, N. Grigorieff, CTFFIND4: Fast and accurate defocus estimation from electron micrographs. *J. Struc. Biol.* **192**, 216–221 (2015).

54. Y. Z. Tan, P. R. Baldwin, J. H. Davis, J. R. Williamson, C. S. Potter, B. Carragher, D. Lyumkis, Addressing preferred specimen orientation in single-particle cryo-EM through tilting. *Nat. Methods* **14**, 793–796 (2017).

55. L. Rohlin, D. R. Leon, U. Kim, J. A. Loo, R. R. Ogorzalek Loo, R. P. Gunsalus, Identification of the major expressed S-layer and cell surface-layer-related proteins in the model methanogenic

archaea: *Methanosarcina barkeri* Fusaro and *Methanosarcina acetivorans* C2A. *Archaea* **2012**, 873589 (2012).

56. J. Erde, R. R. Loo, J. A. Loo, Improving proteome coverage and sample recovery with enhanced FASP (eFASP) for quantitative proteomic experiments. *Methods Mol. Biol.* **1550**, 11–18 (2017).

57. F. Madeira, M. Pearce, A. R. N. Tivey, P. Basutkar, J. Lee, O. Edbali, N. Madhusoodanan, A. Kolesnikov, R. Lopez, Search and sequence analysis tools services from EMBL-EBI in 2022. *Nucleic Acids Res.* **50**, W276–W279 (2022).

58. M. Mirdita, K. Schutze, Y. Moriwaki, L. Heo, S. Ovchinnikov, M. Steinegger, ColabFold: Making protein folding accessible to all. *Nat. Methods* **19**, 679–682 (2022).

59. S. J. Ludtke, P. R. Baldwin, W. Chiu, EMAN: Semiautomated software for high-resolution single-particle reconstructions. *J. Struc. Biol.* **128**, 82–97 (1999).

60. P. V. Afonine, B. K. Poon, R. J. Read, O. V. Sobolev, T. C. Terwilliger, A. Urzhumtsev, P. D. Adams, Real-space refinement in PHENIX for cryo-EM and crystallography. *Acta Crystallogr. D Struct. Biol.* **74**, 531–544 (2018).

61. E. F. Pettersen, T. D. Goddard, C. C. Huang, E. C. Meng, G. S. Couch, T. I. Croll, J. H. Morris, T. E. Ferrin, UCSF ChimeraX: Structure visualization for researchers, educators, and developers. *Protein Sci.* **30**, 70–82 (2021).

62. T. Rodrigues-Oliveira, A. A. Souza, R. Kruger, B. Schuster, S. Maria de Freitas, C. M. Kyaw, Environmental factors influence the Haloferax volcanii S-layer protein structure. *PLOS ONE* **14**, e0216863 (2019).
